# Exploring systemic RNA interference in insects: a genome-wide survey for RNAi genes in *Tribolium*

**DOI:** 10.1186/gb-2008-9-1-r10

**Published:** 2008-01-17

**Authors:** Yoshinori Tomoyasu, Sherry C Miller, Shuichiro Tomita, Michael Schoppmeier, Daniela Grossmann, Gregor Bucher

**Affiliations:** 1 Division of Biology, Kansas State University, Manhattan, Kansas 66506, USA; 2 K-State Arthropod Genomics Center, Kansas State University, Manhattan, Kansas 66506, USA; 3 Insect Genome Research Unit, National Institute of Agrobiological Sciences, 1-2, Owashi, Tsukuba, Ibaraki 305-8634, Japan; 4 Universitat Erlangen, Institut fur Biologie, Abteilung fur Entwicklungsbiologie, Staudtstr., D-91058 Erlangen, Germany; 5 Johann-Friedrich-Blumenbach-Institut für Zoologie und Anthropologie, Georg-August-Universität Göttingen, Abteilung Entwicklungsbiologie, Justus-von-Liebig-Weg, 37077 Göttingen, Germany

## Abstract

**Background:**

RNA interference (RNAi) is a highly conserved cellular mechanism. In some organisms, such as *Caenorhabditis elegans*, the RNAi response can be transmitted systemically. Some insects also exhibit a systemic RNAi response. However, *Drosophila*, the leading insect model organism, does not show a robust systemic RNAi response, necessitating another model system to study the molecular mechanism of systemic RNAi in insects.

**Results:**

We used *Tribolium*, which exhibits robust systemic RNAi, as an alternative model system. We have identified the core RNAi genes, as well as genes potentially involved in systemic RNAi, from the *Tribolium *genome. Both phylogenetic and functional analyses suggest that *Tribolium *has a somewhat larger inventory of core component genes than *Drosophila*, perhaps allowing a more sensitive response to double-stranded RNA (dsRNA). We also identified three *Tribolium *homologs of *C. elegans sid-1*, which encodes a possible dsRNA channel. However, detailed sequence analysis has revealed that these *Tribolium *homologs share more identity with another *C. elegans *gene, *tag-130*. We analyzed *tag-130 *mutants, and found that this gene does not have a function in systemic RNAi in *C. elegans*. Likewise, the *Tribolium sid-*like genes do not seem to be required for systemic RNAi. These results suggest that insect *sid-1*-like genes have a different function than dsRNA uptake. Moreover, *Tribolium *lacks homologs of several genes important for RNAi in *C. elegans*.

**Conclusion:**

Although both *Tribolium *and *C. elegans *show a robust systemic RNAi response, our genome-wide survey reveals significant differences between the RNAi mechanisms of these organisms. Thus, insects may use an alternative mechanism for the systemic RNAi response. Understanding this process would assist with rendering other insects amenable to systemic RNAi, and may influence pest control approaches.

## Abstract 2

Tribolium resembles C. elegans in showing a robust systemic RNAi response, but does not have C. elegans-type RNAi mechanisms; insect systemic RNAi probably uses a different mechanism.

## Background

A decade has passed since the discovery that double-stranded RNA molecules (dsRNA) can trigger silencing of homologous genes, and it is now clear that RNA-mediated gene silencing is a widely conserved cellular mechanism in eukaryotic organisms [1-3]. RNA-mediated gene silencing can be categorized into two partially overlapping pathways; the RNA interference (RNAi) pathway and the micro-RNA (miRNA) pathway [2,4-6]. RNAi is triggered by either endogenous or exogenous dsRNA, and silences endogenous genes carrying homologous sequences at both the transcriptional and post-transcriptional levels. In contrast, the miRNA pathway is triggered by mRNAs transcribed from a class of non-coding genes. These mRNAs form hairpin-like structures, creating double-stranded regions in a molecule (pre-miRNA). In either pathway, dsRNA molecules are processed by Dicer RNase III proteins into small RNAs (for a review of Dicer, see [7]), which are then loaded into silencing complexes (reviewed in [8]). In the RNAi pathway, small RNAs are called short interfering RNAs (siRNAs) and are loaded into RNA-induced silencing complexes (RISC) for post-transcriptional silencing, or RNA-induced initiation of transcriptional gene silencing (RITS) complexes for transcriptional silencing. In contrast, miRNAs (small RNAs in the miRNA pathway) are loaded into miRNA ribonucleoparticles (miRNPs) (see [2] for a review of silencing complexes). dsRNA binding motif (dsRBM) proteins, such as R2D2 and Loquacious, help small RNAs to be loaded properly into silencing complexes [9-14]. Using the small RNA as a guide, silencing complexes find target mRNAs and cleave them (in the case of RISC) or block their translation (in the case of miRNP). RITS is involved in transcriptional silencing by inducing histone modifications. Argonaute family proteins are the main components of silencing complexes, mediating target recognition and silencing (reviewed in [15,16]). The RNAi pathway and miRNA pathway are essentially parallel, using related but distinct proteins at each step. For instance, in *Drosophila*, Dicer2, R2D2 and Argonaute2 are involved in the RNAi pathway, while Dicer1, Loquacious, and Argonaute1 function in the miRNA pathway [10,12,14,17,18]. In *Caenorhabditis elegans*, the primary siRNAs processed by Dicer are used as guides for RNA-dependent RNA polymerase (RdRP) to produce secondary dsRNAs in a two-step mechanism [19,20]. This amplification step is apparently essential for the RNAi effect in *C. elegans *[19-21].

RNAi has become a widely used tool to knock down and analyze the function of genes, especially in non-model organisms where the systematic recovery of mutants is not feasible. However, in some organisms, delivery of dsRNA presents a problem. Injecting dsRNA directly into eggs seems to be the most efficient way to induce an RNAi effect; however, many embryos do not survive the injection procedure, the number of knock-down embryos generated is limited and all individuals have been injured by the injection. In addition, in some species such as *Drosophila*, dsRNA injection into embryos sometimes results in a mosaic pattern of knock-down effect [22]. Furthermore, knocking down genes frequently kills the embryo, making it difficult to perform functional analyses of these genes at later, post-embryonic stages. In a few highly established model systems, such as *Drosophila*, hairpin constructs can be used to overexpress dsRNA in particular tissues at certain stages [23-25]. Virus-mediated methods offer an alternative way to overexpress dsRNA [26]; however, some organisms seem to eliminate virus quickly (M Jindra, personal communication), making it difficult to apply this method globally. In some organisms (but not others) dsRNA can be introduced at postembryonic stages by feeding, soaking or direct injection (for example, larval/nymphal stage [27-31], adult stage [32-37], feeding RNAi [38,39], soaking RNAi [40]). The dsRNA somehow enters cells and induces an RNAi effect systemically. Transmission of the RNAi effect to the next generation is also possible (parental RNAi [41-45]). However, some organisms, such as the silkworm moth *Bombyx mori*, do not show a robust systemic RNAi response [46] (ST, unpublished data; R Futahashi and T Kusakabe, personal communications; but see also [47-49] for some successful cases). Understanding the molecular mechanisms underlying systemic RNAi may help in applying RNAi techniques to these organisms.

Systemic RNAi was first described in plants as spread of post-transcriptional gene silencing [50-52]. The first animal in which RNAi was shown to work systemically was *C. elegans*, where it has been thoroughly investigated [1,53] (for reviews of systemic RNAi, see [54-57]). The phenomenon can be subdivided into two distinct steps: uptake of dsRNA by cells, and systemic spreading of the RNAi effect [58]. Several genes have been identified in *C. elegans *as important for systemic spread but not for the interference itself. *sid-1 *encodes a multi-transmembrane domain protein, which is thought to act as a channel for dsRNA [53,59]. Mosaic analysis in *C. elegans *as well as the overexpression of Sid-1 in cultured cells show that Sid-1 is involved in the dsRNA uptake step in both somatic and germ-line cells [53,59]. Three more proteins, Rsd-2, Rsd-3, and Rsd-6, have been identified as important factors for the systemic RNAi response in germ-line but not somatic cells [60]. Recently, over 20 genes have been reported to be necessary for dsRNA uptake in *Drosophila *tissue culture cells [61,62]. Many of the genes identified in this system have been previously implicated in endocytosis, suggesting that this process may play an important role in dsRNA uptake also in other cells [61,62].

Interestingly, core RNA machineries are not involved in systemic RNAi spreading in *C. elegans*. Homozygous Argonaute mutant (*rde-1*) individuals are still capable of transmitting the RNAi effect from intestine to gonad [63]. The same result is observed in *rde-4 *mutants (*rde-4 *encodes a dsRBM protein that acts upstream of Rde-1) [63]. These mutants produce only initial siRNAs, which represent only a trace amount compared to the secondary siRNAs and are not sufficient to trigger any RNAi response [21,64]. These data indicate that, at least in these mutant conditions, siRNA production and amplification are not necessary for spreading of the RNAi effect in *C. elegans*, suggesting that dsRNA itself may be the transmitting factor for RNAi spreading. Longer dsRNA is preferably imported by tissue culture cells overexpressing the *C. elegans sid-1 *gene, which supports this view [59]. Moreover, 50 bp dsRNA injected into an intestinal cell is too short to induce systemic RNAi in *C. elegans *[59], suggesting that it is not siRNAs or dsRNA subsequently produced by RdRP, but rather the long initial dsRNA, which is critical for the systemic RNAi response.

Although, systemic RNAi spreading from cell to cell has not been shown in any animals other than *C. elegans *(spreading does not seem to occur in *Drosophila *([65]), systemic uptake of dsRNA by cells seems to be conserved in some insects [27-30,32-37,41,42,45]. Unfortunately, the systemic aspect of RNAi in *Drosophila*, the prime insect model organism, has not been studied thoroughly, and the extent to which systemic RNAi occurs in this insect is still unknown. Some tissues in *Drosophila *adults (including oocytes) [35,36,45] seem to be capable of taking up dsRNA; however, the systemic RNAi response seems to be greatly reduced in the larval stage (SCM and YT, unpublished data). In addition, parental RNAi at the pupal stage for some genes has failed (GB and M Klingler, unpublished data). The lack of a robust systemic RNAi response in *Drosophila *necessitates another model system if systemic RNAi is to be studied in insects. The red flour beetle, *Tribolium castaneum*, is the best characterized insect genetic model system besides *Drosophila*. Since *Tribolium *has the ability to respond to dsRNA systemically [27,41], it is an ideal model system for studying this process in insects.

The recently completed genomic sequence of *T. castaneum *[66] allowed us to comprehensively analyze the inventory of *Tribolium *homologs of genes involved in RNA-mediated gene silencing and the systemic RNAi response. Our results suggest that the molecular mechanisms for both RNAi amplification and dsRNA uptake in *Tribolium *are different from those in *C. elegans*. Therefore, systemic RNAi in insects might be based on a different mechanism that remains to be discovered. We also noticed several differences in the number of RNAi core component genes between *Tribolium *and *Drosophila*. These differences might contribute to the robust RNAi response in *Tribolium*. Based on our results we discuss several factors that might make *Tribolium *so amenable to systemic RNAi.

## Results

### Core RNAi components

Although the core components of RNA-mediated gene silencing are usually well conserved among species, the number and the degree of conservation of these proteins often vary between species. The efficiency of RNAi might affect the degree of systemic RNAi response. Therefore, we have surveyed genes that encode some core RNAi components.

#### Dicer and dsRBM protein family

**Figure 1:**
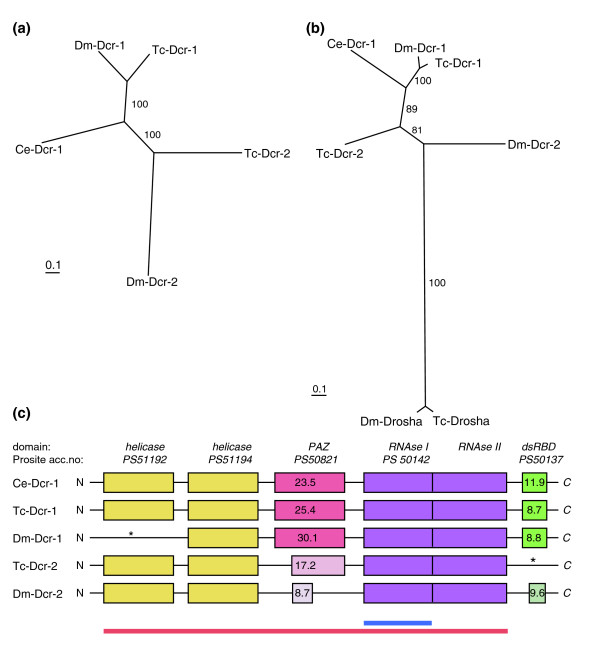
Phylogenetic and protein domain analysis of Dicer proteins. **(a, b) **Phylogenetic analysis of Dicer proteins (a) and with Drosha as an outgroup (b). The tree in (a) was composed based on the alignments of full-length Dicer proteins without dsRBD (c, red underline), while the tree in (b) was based on the RNase I domain (c, blue underline). The *Drosophila *and *Tribolium *Dcr-1 proteins cluster together, indicating clear orthology. In contrast, orthology of Dcr-2 proteins in these insects is less clear since they do not cluster together. **(c) **Domain architecture of Dicer proteins. Although our phylogenetic analysis cannot solve the orthology of insect Dcr-2 proteins, the similarity in the domain architectures of Dm-Dcr-2 and Tc-Dcr-2 suggests that they might be orthologous. Tc-Dcr-1 has a similar domain architecture to Ce-Dcr-1, which is involved both in RNAi and miRNA pathways, suggesting that Tc-Dcr-1 might also be involved in both pathways (unlike Dm-Dcr-1, which is involved only in the RNAi pathway). The ScanProsite scores are shown and the location of domain truncations is indicated. The first helicase domain in Dm-Dcr-1 and dsRBD in Tc-Dcr-2 (indicated by an asterisk) are not recognized by ScanProsite but some conserved residues are identified by ClustalW alignment.

Dicer family proteins are involved in the production of small RNA molecules and have several conserved motifs (Figure 1c) [7,67]: two amino-terminal DExH-Box helicase domains, a PAZ (Piwi/Argonaute/Zwille) domain, tandem RNase III domains and a carboxy-terminal dsRNA binding domain. A single Dicer protein is involved in both the siRNA and miRNA pathways in C. elegans [67-69]. In contrast, different Dicer proteins are assigned to the siRNA and miRNA pathways in Drosophila [17]. Dcr-1, which retains a PAZ domain but lacks an amino-terminal helicase domain (Figure 1c), is involved in the miRNA pathway [17]. On the other hand, Dcr-2 seems to lack a full-length PAZ domain but has the helicase domain (Figure 1c), and is involved in the RNAi pathway [17]. In addition, a distantly related RNase III emzyme, Drosha, is involved in the maturation of miRNA precursors [70,71].

We identified one *drosha *and two Dicer genes in the *Tribolium *genome. One gene (*Tc-Dcr-1*) clearly codes for the ortholog of Dm-Dcr-1 and Ce-Dcr-1. The sequence of the second *Tribolium *Dicer does not clearly cluster with Dm-Dcr-2 (Figure 1a, b). However, as it shares some similarities in domain architecture with Dm-Dcr-2 (Figure 1c, and see below), we tentatively call it Tc-Dcr-2.

A ScanProsite search [72] has revealed that, in contrast to Dm-Dcr-1, which lacks a helicase domain, Tc-Dcr-1 retains both the helicase and PAZ domains (Figure 1c). This domain architecture makes Tc-Dcr-1 more similar to Ce-Dcr-1. Tc-Dcr-2 also has both domains, but the PAZ domain is more diverged (Figure 1c). ScanProsite shows high scores for the PAZ domains of Ce-Dicer-1, Tc-Dcr-1, and Dm-Dcr-1 (scores of 24, 23 and 30, respectively), while the PAZ domain in Tc-Dcr-2 shows a lower score (score 17) (see Materials and methods for a brief explanation of these scores). Dm-Dcr-2, which lacks a full-length PAZ domain, shows a much lower score for the PAZ domain region (score 8). Tc-Dcr-2 also lacks the carboxy-terminal dsRNA binding domain. The diverged PAZ domain and the lack of the dsRNA binding domain make Tc-Dcr-2 more similar to Dm-Dcr-2 (Figure 1c).

A group of dsRBM-containing proteins act with Dicer to load small RNA molecules into a silencing complex. In *Drosophila*, each Dicer protein acts with a particular dsRBM protein: Loquacious (Loqs) for Dcr-1, R2D2 for Dcr-2, and Pasha for Drosha [10-14,73]. Interestingly, these proteins seem to determine the specificity of Dicer proteins, since *Drosophila *Dcr-1, which normally processes miRNAs, can instead produce siRNA in a *loqs *mutant [11,14]. This suggests that differences in these dsRBM-containing proteins might affect the efficiency of RNAi in different organisms.

**Figure 2:**
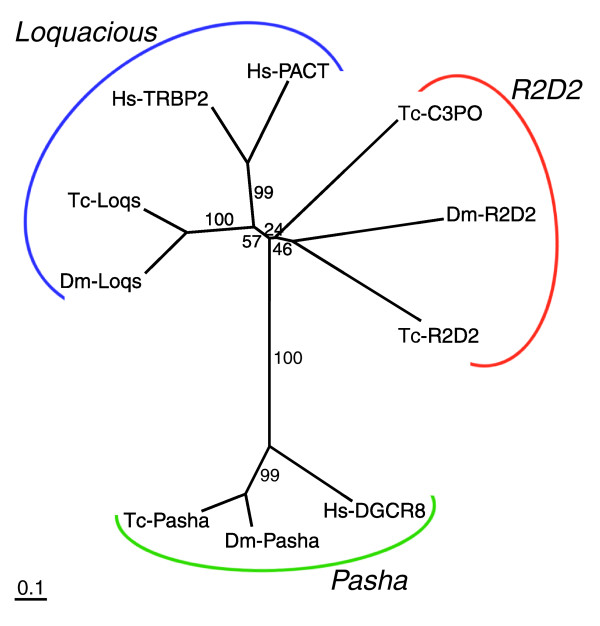
Phylogenetic analysis of dsRBM proteins. The neighbor-joining tree is based on alignment of the tandem dsRBM domains. The *Tribolium *genome contains two R2D2-like proteins (Tc-R2D2 and Tc-C3PO) while *Drosophila *has only one. PACT [135], TRBP2 [136,137], and DGCR8 [138] were included as human counterparts.

We found clear orthologs of *Drosophila loqs *and *pasha *in *Tribolium *(Figure 2). In contrast, the *Tribolium *genome contains two R2D2-like genes (we named one of them *Tc-R2D2 *and the other *Tc-C3PO*), but orthology with *Drosophila *R2D2 is not as clear as for the other dsRBM proteins (Figure 2).

In conclusion, *Drosophila *and *Tribolium *have the same number of Dicer proteins. However, similarity of domain architecture of Tc-Dcr-1 to Ce-Dcr-1 (rather than to Dm-Dcr-1) suggests that, in addition to Tc-Dcr-2, Tc-Dcr-1 could also be involved in both the miRNA and RNAi pathways, perhaps contributing to the robust RNAi response in *Tribolium*. The presence of an additional R2D2-like protein might also help make *Tribolium *hypersensitive to dsRNA molecules taken up by cells.

#### Argonaute family

Argonaute proteins are core components of RISC and miRNP, and are involved in siRNA-based as well as miRNA-based silencing [2,16]. Some Argonaute proteins are also involved in transcriptional silencing as a component of RITS [74,75]. Different Argonaute proteins are used for each process [16]. For instance, in *Drosophila*, Ago-1 and Ago-2 are predominantly used for miRNA and siRNA pathways, respectively [18], while Piwi, Aubergine (Aub), and Ago-3 are used for transcriptional silencing [76-79]. Argonaute proteins contain two distinctive domains: a PAZ domain and a PIWI domain [16]. The PAZ domain seems to be involved in dsRNA binding, while the PIWI domain possesses RNase activity.

There is a striking expansion of Argonaute proteins in *C. elegans *(27 Argonaute proteins have been identified) [80]. As in *Drosophila*, these Argonaute proteins function in different processes. Rde-1 and Ergo-1 have been identified to act in the RNAi pathway [9,80], while Alg-1 and Alg-2 are important for the miRNA pathway [81]. Yigit *et al*. [80] identified yet another class of Argonaute proteins, the secondary Argonautes (Sago), that interact specifically with the siRNAs produced via RdRP amplification but not with the initial siRNAs. These results led the authors to propose a two-step model: first, the primary siRNAs, which are produced from the initial dsRNA, bind specifically to the initial Argonautes (Rde-1 or Ergo-1), and second, subsequent amplification by RdRP leads to the production of secondary siRNAs, which exclusively bind to secondary Argonaute proteins. This two-step recognition is proposed to be required for amplification of the RNAi effect, and at the same time possibly reducing off-target effects. As the secondary Argonaute proteins lack critical metal binding residues in the catalytic RNAse H-related PIWI domain, they are predicted to recruit other nucleases for degradation of target mRNAs [80].

**Figure 3:**
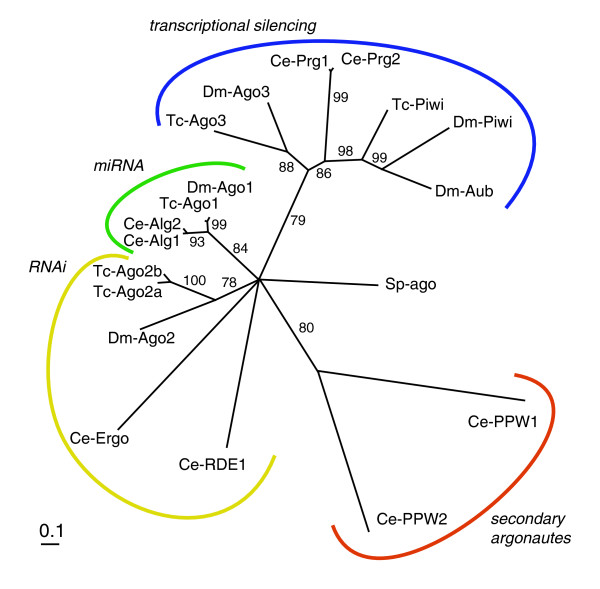
Phylogenetic analysis of Argonaute proteins. The neighbor-joining tree is based on the alignment of the conserved PIWI domain. Argonaute proteins can be categorized into four groups, each important for a different process; the RNAi pathway, the miRNA pathway, transcriptional silencing, and amplification of the RNAi effect (secondary Argonautes). *Tribolium *and *Drosophila *lack secondary Argonautes, suggesting that the secondary Argonaute-based amplification mechanism is not conserved in these insects.

Both *Tribolium *and *Drosophila *have five Argonaute genes. To investigate the orthology relationships of these genes we calculated a tree based on an alignment of the PIWI domains of all *Tribolium *and *Drosophila *Argonaute proteins, a representative selection of *C. elegans *paralogs and the single *Schizosaccharomyces pombe *Argonaute protein (Figure 3; see Additional data file 1 for the alignment).

A single miRNA class Argonaute (Ago-1 in *Drosophila *and Alg-1/Alg-2 in *C. elegans*) is present in *Tribolium *(Tc-Ago-1). For the siRNA class Argonautes, we found two Ago-2 paralogs in *Tribolium *(Tc-Ago-2a and Tc-Ago-2b) that probably stem from a duplication in the lineage leading to beetles. These two proteins are clearly orthologous to *Drosophila *Ago-2; however, the relationships to *C. elegans *Rde-1 and Ergo-1 are not resolved in our analysis. The duplication of Ago-2 in *Tribolium *might lead to higher amounts of Tc-Ago2 protein and, hence, an enhanced RNAi response.

For the Piwi/Aub class Argonautes, which are involved in transcriptional silencing, we find one *Tribolium *ortholog (Tc-Piwi) of the *Drosophila *Piwi and Aub. One additional protein of this family (Tc-Ago3) is orthologous to a recently described *Drosophila *protein, Dm-Ago3 [77,82]. All these insect PIWI-type proteins are orthologous to the *C. elegans *Prg-1 and Prg-2.

Importantly, we do not find any homologs of secondary Argonaute proteins (represented by Ce-Ppw-1 and Ppw-2 in our tree) in either *Tribolium *or *Drosophila *(Figure 3). Furthermore, we confirmed that all *Tribolium *and *Drosophila *Argonaute proteins do have the metal binding residues of the PIWI domain, unlike the *C. elegans *secondary Argonaute proteins, which lack them [80]. The only exception is *Drosophila *Piwi, which has a lysine instead of a histidine in the third position. These data, along with the fact that the *Tribolium *genome lacks an ortholog of RdRP (see below), suggest that the two-step RNAi mechanism of RdRP-mediated amplification followed by secondary Argonaute function is not conserved in either *Tribolium *or *Drosophila*. The different abilities of *Drosophila *and *Tribolium *to perform systemic RNAi might, therefore, depend on factors other than the Argonaute repertoire in these insects.

#### Absence of RNA-dependent RNA polymerase in *Tribolium*

**Figure 4:**
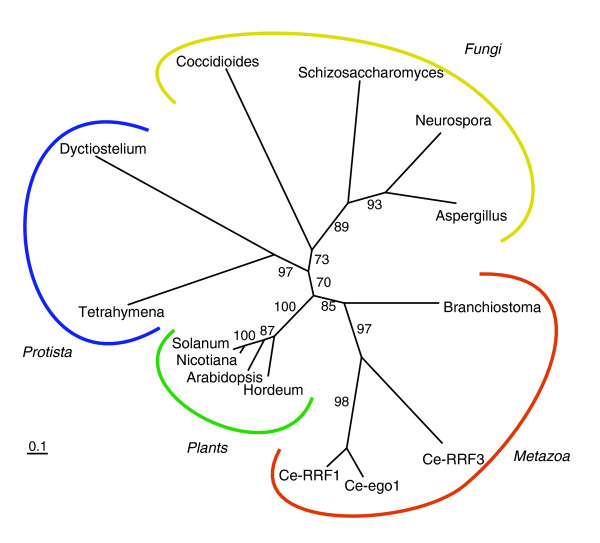
Distribution of RdRP in eukaryotes. Although RdRPs are present in many plants, fungi and protists (a selection is included in this tree), of the Metazoa, only *Caenorhabditid *nematodes and a chordate *Branchiostoma *are found to carry RdRP genes. Plant and protist RdRPs cluster together with very high support, while fungus and animal RdRPs comprise distinct clusters. *Caenorhabditid *RdRPs are represented by the three *C. elegans *paralogs RRF-1/3 and Ego-1. Species names of the organisms shown in this tree are as follows: animals, *Branchiostoma floridae*; fungi, *Coccidioides immitis*, *S. pombe*, *Neurospora crassa *and *Aspergillus terreus*; plants,*Hordeum vulgare*, *Arabidopsis thaliana*, *Nicotiana tabacum *and *Solanum lycopersicum*; protists, *Dictyostelium discoideum *and *Tetrahymena thermophila*.

Systemic RNAi relies on the distribution of the trigger dsRNA, its uptake and subsequent efficient gene knockdown in cells. The distribution of the dsRNA trigger leads to its dilution [83]. Hence, a mechanism for enhancing the signal may be required for efficient silencing. RdRP is a key for the amplification of the RNAi effect in *C. elegans *as well as in several plants [19,20,84,85]. It is possible that *Tribolium *has a similar amplification mechanism. However, we do not find a gene encoding an RdRP-related protein in the *Tribolium *genome by BLAST searches. Moreover, a BLAST search of all metazoan genes in the NCBI database identified RdRP genes only in several *Caenorhabditis *species and a cephalochordate *Branchiostoma floridae *[86]. Even some nematode species outside *Caenorhabditis *do not seem to carry RdRP genes. All other eukaryotic RdRPs belong to plants, fungi or protists, suggesting that RdRP is not conserved in animals (Figure 4). The lack of an RdRP gene in *Tribolium *suggests that the strong RNAi response in *Tribolium *does not rely on amplification of the trigger dsRNA by RdRP.

#### Eri-1-like exonuclease family

In *C. elegans*, several tissues, such as the nervous system, are refractory to RNAi, apparently due to the expression of *eri-1 *[87]. Abundant siRNA accumulates in *eri-1 *mutants, suggesting that Eri-1 is involved in siRNA degradation [87]. The *eri-1 *gene encodes an evolutionarily conserved protein that contains a SAP/SAF-box domain and DEDDh family exonuclease domain [87]. The expression level and/or tissue specificity of *eri-1 *homologs might cause differences in sensitivity to dsRNA among organisms.

**Figure 5:**
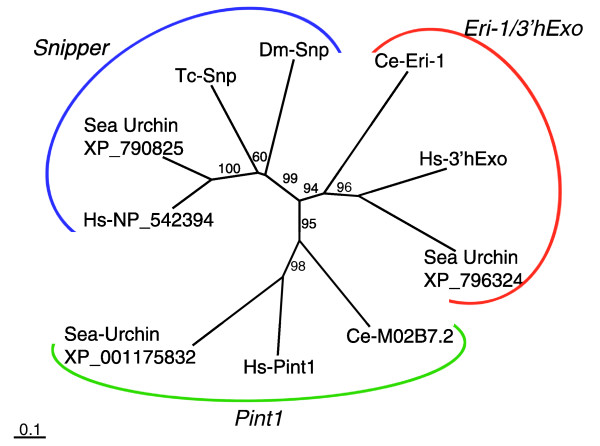
Phylogenetic analysis of Eri-1-like exonucleases. The neighbor-joining tree is based on the alignment of the exonuclease domain. Eri-1-like nucleases cluster into three subclasses: Eri-1/3'Exo, Snipper, and Pint1. *Tribolium *and *Drosophila *have only Snipper-type nucleases. One human and three sea urchin (*Strongylocentrotus purpuratus*) proteins are represented by NCBI accession numbers.

We have identified an *eri-1*-like gene in *Tribolium*. 5' and 3' rapid amplification of cDNA ends (RACE) analysis has revealed that this gene encodes a 232 amino acid protein (see Materials and methods for details). We also found a close homolog of this gene in *Drosophila *(CG6393, *Dm-snipper*). Interestingly, these genes are lacking the amino-terminal SAP/SAF-box domain. Also, phylogenetic analysis using the nuclease domain (Additional data file 1) reveals that the insect homologs cluster together, while Ce-Eri-1 and its human ortholog (3'hExo; three prime histone mRNA exonuclease [88]) compose another subclass. We subsequently noticed that there are at least three subclasses of nucleases closely related to Eri-1 in metazoans: the Eri-1/3'hExo subclass, the Pint1 (Prion Interactor 1 [89], also named Prion protein interacting protein (PrPIP) in [90]) subclass, and the Snipper subclass (Figure 5). Humans as well as sea urchins have all three subclasses of nucleases. *C. elegans *has at least two types of these nucleases, which belong to the Eri-1/3'hExo and Pint1 subclassses, respectively. In addition, it contains another nuclease (Cell-death-related nuclease 4 (Crn-4) [91]), whose position relative to the three subclasses of nucleases is unclear. Crn-4 clusters with *C. elegans *Eri-1 (Additional data file 2), but this affinity is questionable since Crn-4 does not share the amino-terminal region that is conserved in other members of the Eri-1/3'Exo subclass. The *Tribolium *and *Drosophila *nucleases, with their vertebrate and sea urchin orthologs, compose a distinct subclass (Snipper subclasss). This suggests that *Drosophila *and *Tribolium *lack nucleases belonging to the Eri-1 subclass, and that the insect nucleases might have a function other than siRNA digestion.

Recently, the *Drosophila *nuclease has been characterized as Snipper (Snp) [90]; therefore, we have named the *Tribolium *ortholog Tc-Snp. Although Snp can cleave RNA as well as DNA molecules *in vitro*, Snp seems to have no role in RNAi in *Drosophila *[90]. This supports our idea that the Snp subclass nucleases might not have an important role in the RNAi pathway. In conclusion, it is unlikely that nucleases related to Eri-1 are causing the differential sensitivity to dsRNA in *Tribolium *and *Drosophila*.

### Candidate factors for systemic RNAi in *Tribolium*

Several proteins are important for the systemic spread of the RNAi response in *C. elegans *but not for the RNAi pathway itself [53,60]. However, the degree of conservation of these proteins in other organisms has not been described. The presence of these factors might be critical for robust systemic RNAi. In addition, dozens of proteins have recently been identified as crucial for dsRNA uptake in *Drosophila *S2 cells [61,62]. We have screened the *Tribolium *genome for homologs of both of these groups of proteins.

#### Sid-1-like proteins

Sid-1 is the best characterized protein involved in systemic RNAi in *C. elegans *[53,59]. The Sid-1 protein contains a long amino-terminal extracellular domain followed by an array of transmembrane domains, which are inferred to form a channel for dsRNA molecules [53,59]. Mosaic analysis in *C. elegans *using a *sid-1 *overexpression construct showed that Sid-1 is cell-autonomously required for receiving the systemic RNAi signal (it is still possible that Sid-1 is also involved in the RNAi spreading step) [53]. Overexpression of *sid-1 *in *Drosophila *culture cells also enhances the ability of the cells to uptake dsRNA from the culture media, further suggesting an important role for Sid-1 in dsRNA uptake [59]. *C. elegans *carries two additional *sid-1 *like genes, *tag-130 *(also known as ZK721.1) and Y37H2C1, although their functions are unclear. Many vertebrate species also have *sid-1 *homologs [53,92]. However, *Drosophila*, which does not show a robust systemic RNAi response, lacks *sid-1*-like genes, leading to the hypothesis that the presence or absence of a *sid-1-like *gene is the primary determinant of whether or not systemic RNAi occurs in an organism [28,53,92-94].

We have identified three *sid-1*-like genes in the *Tribolium *genome. We have decided to call these genes *sil *(*sid1-like; Tc-silA-C*) instead of *Tc-sid-1*, because of uncertainty about the orthology of insect *sid1-like *genes to *C. elegans sid-1 *(see below). RT-PCR and RACE analyses have revealed the full-length sequences (Tc-SilA, 764 amino acids; Tc-SilB, 732 amino acids; Tc-SilC, 768 amino acids, see Materials and methods for details). Like *C. elegans *Sid-1, all three proteins contain a long amino-terminal extracellular domain followed by 11 transmembrane domains predicted by TMHMM server version 2.0. InterProScan identified no additional motifs or domains.

To determine whether the presence of *sil *genes correlates with the presence of systemic RNAi in insects, we have searched the genome of several insects using the Tc-SilA protein sequence as a query (Table 1). The honeybee (*Apis mellifera*; Hymenoptera) and a parasitic wasp (*Nasonia vitripennis*; Hymenoptera) each contain a single *sid-1*-like gene. The silkworm moth (*B. mori*; Lepidoptera) has three *sid-1*-like genes. We have determined the full-length sequences of these genes in *Bombyx *(see details in Materials and methods). As previously mentioned, *D. melanogaster *does not have any *sid-1*-like genes. We have confirmed that none of the 11 additional *Drosophila *species whose genomes have been sequenced carry *sid-1 *family genes. In addition, two mosquito species (*Anopheles gambiae *and *Aedes aegypti*) also lack *sid-1*-like genes, suggesting the early loss of *sid-1*-like genes in the dipteran lineage.

**Table 1:** Incidence of *sil *genes and systemic RNAi in insects

		Systemic RNAi			
			
Species	*sil *gene number	Larval/nymphal	Adult	Parental	References
*Drosophila melanogaster*	0	ND*	Some tissues^†^	Yes	[35,36,44]
12 Drosophilids	0	ND	ND	ND	
*Anopheles gambiae*	0	ND	Some tissues^†^	No^‡^	[33,34]
*Aedes aegypti*	0	ND	Some tissues^†^	ND	[34,37]
*Bombyx mori*	3	Limited success^§^	ND	ND	[45-48]
*Apis mellifera*	1	Some tissues^†^	Some tissues^†^	ND	[32,38]
*Nasonia vitripennis*	1	ND	ND	Yes	[41]
*Tribolium castaneum*	3	Yes^¶^	Some tissues^†^	Yes	[27,40]
*Schistocerca americana*	≥ 1	Some tissues^†^	ND	ND	[28]

*Yes in hemocyte (SCM and YT, unpublished results). ^†^RNAi has been successfully performed in some tissues (but not in other tissues). ^‡^Ovary can take up dsRNA, but parental RNAi has been unsuccessful (MGorman, personal comunication). ^§^ST, unpublished data, R Futahashi and T Kusakabe, personal communications. ^¶^All tissues are suceptible (SCM and YT, unpublished results). ND, not determined.

The presence of three *sil *genes in *Tribolium *is consistent with their hypothesized importance to a robust systemic RNAi response. It has also been shown that parental RNAi is possible in *Nasonia *[42], which is consistent with the presence of a *sil *gene in this insect. On the surface, the lack of *sid-1*-like genes in dipterans seems to correlate with the apparent lack of systemic RNAi response in these insects. However, reports that some tissues in *Drosophila *as well as in mosquitos are capable of taking up dsRNA [33-37,45] (MJ Gorman, personal communication) suggest that such correlations might be misleading. Moreover, *Bombyx *carries three *sil *genes, yet does not show a robust systemic RNAi response (S Tomita, unpublished data; R Futahashi and T Kusakabe, personal communications). This apparent breakdown in the correlation between systemic RNAi and *sil *genes (Table 1) raises the question of whether *sid-1*-like genes are the determinant of presence/absence of systemic RNAi in insects.

We have analyzed the expression of *sil *genes to provide a clue about the function of these genes in *Tribolium*. *in situ *hybridization analysis shows that all three *sil *genes are expressed uniformly in embryos; however, *silA *and *silB *seem to be expressed at lower levels than *silC *(data not shown). Semi-quantitative RT-PCR reveals that all *sil *genes are expressed throughout all developmental stages (Additional data file 3). *silA *and *silB *expression level is uniform through the larval to adult stages, while *silC *has peak expression at the pupal stage.

**Figure 6:**
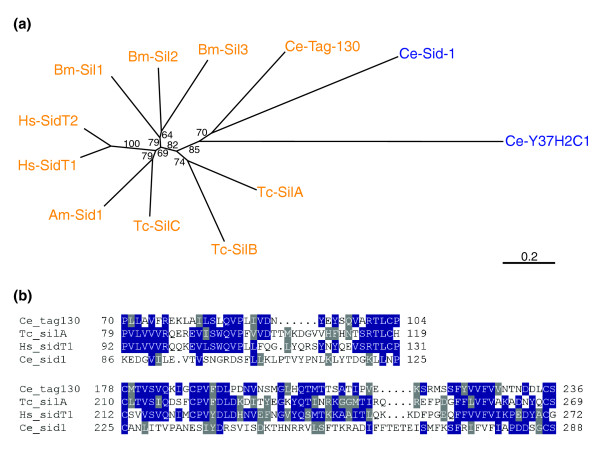
Sil protein alignment and phylogenetic analysis. **(a) **Phylogenetic analysis of Sid-1-like proteins. The neighbor-joining tree is based on the alignment of the carboxy-terminal transmembrane domain corresponding to the TM2-TM11 region of *C. elegans *Sid-1 (Additional data files 1 and 4). Tc-SilC clusters with the human Sid-1-like proteins (SidT1 and SidT2), while Tc-SilA and Tc-SilB compose a distinct cluster. Orthology of these insect and vertebrate Sid-1-like proteins to the *C. elegans *homologs is unclear from this analysis. Proteins that contain the amino-terminal conserved region are indicated in red. **(b) **Two conserved regions in the amino-terminal extracellular domain. These regions are conserved in vertebrate Sid-1-like proteins (represented by human SidT1), insect Sil proteins (Tc-SilA), and *C. elegans *Tag-130, but not in *C. elegans *Sid-1.

We have performed phylogenetic analyses using the carboxy-terminal conserved region (the region corresponding to the second to tenth transmembrane domains; Additional data file 4) to solve the orthology of Sid1-like proteins. Both neighbour-joining and maximum-likelihood analyses produce the same tree with slightly different bootstrap values (see Figure 6a for the neighbour-joining tree). In these trees, all three *C. elegans *proteins comprise a distinct cluster. Two of the *Tribolium *Sil proteins (Tc-SilA and Tc-SilB) also comprise a separate cluster, while Tc-SilC clusters with honeybee as well as vertebrate Sid-1-like proteins. Bombyx Sil proteins belong to this cluster; however, they comprise a distinct sub-cluster in this branch. This result is somewhat puzzling since it appears to suggest multiple occurrences of lineage-specific duplication. Alternatively, the expansion of *sil *genes might be ancient, but the paralogs might have been subjected to lineage specific parallel constraints (perhaps to target a species specific ligand), leading to convergent sequence similarity. The clustering of the three *C. elegans *homologs might be due to a long branch attraction caused by their highly diverged sequences. The clustering of vertebrate Sid-like proteins with Tc-SilC and the honeybee proteins might suggest a conserved function in this cluster.

Although the carboxy-terminal transmembrane region shows a high degree of identity between all Sid-1-like proteins, the amino-terminal extracellular region is less conserved (Additional data files 4 and 5). We noticed, however, that there are several segments in the extracellular region that are shared by insect and vertebrate Sid-1-like proteins (Figure 6b; see also Additional data file 5 for dot-matcher alignments). Interestingly, *C. elegans *Tag-130, but not Sid-1, also shares these amino-terminal motifs (Figure 6a, Additional data file 5), raising questions about the orthology of insect/vertebrate Sid-like proteins and *C. elegans *Sid-1. Sil proteins in insects and vertebrates might instead be orthologous to *C. elegans *Tag-130.

Although our phylogenetic analysis is inconclusive on the orthology of insect Sil proteins, the sequence similarity of the amino-terminal extracellular region between Sil proteins and *C. elegans *Tag-130 suggests that these proteins may share similar functions. To gain further insight into the function of *sil *genes, we have analyzed whether *tag-130 *has any function in systemic RNAi in *C. elegans*. We obtained two deletion alleles of *tag-130 *from the *Caenorhabditis *Genetics Center. One allele, *tag-130*^*gk*245^, has been described to have a 711 bp deletion that removes the promoter region as well as the first 221 bp of the coding region (73 amino acids) (Additional data file 6). We have confirmed this deletion by PCR. We have also determined that the other allele, *tag-130*^*ok*1073^, has a 689 bp deletion spanning several exons that encode transmembrane domains (exons 14 to 17; see Additional data file 6 for the detailed deleted region). RT-PCR analysis has revealed that *tag-130*^*gk*245 ^lacks *tag-130 *gene transcription, suggesting that this is a null allele. We have detected two different forms of mRNA transcribed in *tag-130*^*ok*1073^, both of which encode truncated proteins (Additional data file 6). These proteins lack several transmembrane domains, suggesting that *tag-130*^*ok*1073 ^is also a null allele. To determine whether these mutants are susceptible to systemic RNAi, we fed them *unc-22 *dsRNA expressing *E. coli*. The N2 wild-type strain was used as a positive control, and *sid-1*^*sq*2^, a null allele for *sid-1 *[53,59], was used as a negative control. If *tag-130 *is involved in systemic RNAi, mutations in the *tag-130 *gene should prevent the *unc-22 *RNAi twitching effect [95]. However, almost 100% of individuals carrying either *tag-130 *deletion allele show a twitching phenotype upon administration of *unc-22 *feeding RNAi, while none of the *sid-1 *individuals showed twitching (Table 2). These data indicate that *tag-130 *is not necessary for the systemic RNAi response in *C. elegans*. By extension, the greater sequence similarity of insect Sil proteins to Tag-130 than to Sid-1 suggests that Sil proteins might not be involved in systemic RNAi in *Tribolium*.

**Table 2:** Feeding RNAi in *sid-1 *and *tag-130 *mutants

Genotype	Total number	*unc-22*	*non-unc*	% *unc *phenotype
N2 (wild type)	315	308	10	97.8%
*tag-130*^*gk*245^	341	340	1	99.7%
*tag-130*^*ok*1073^	140	138	2	98.6%
*sid1*^*sq*2^	297	0	297	0.0%

#### *C. elegans rsd *gene homologs

Another screen for *C. elegans *mutants lacking systemic RNAi led to the discovery of several additional genes involved in the systemic RNAi response, including *rsd-2*, *rsd-3*, and *rsd-6 *[60]. Mutants for these genes still retain the systemic RNAi response in somatic cells, but germ-line cells lack the ability to respond to dsRNA [60]. The Rsd-2 protein contains no particular motifs, while Rsd-6 has a Tudor domain, which is found in some RNA binding proteins [60]. A yeast two-hybrid analysis found that Rsd-2 interacts directly with Rsd-6, suggesting that these proteins act together [60]. We do not find *Tribolium *homologs for *rsd-2 *or *rsd-6 *in the genomic sequence of *Tribolium *(Table 3) or in several other insects whose genomes have been sequenced, which suggests that the Rsd-2/Rsd-6 system is either not conserved in insects, or is evolving too rapidly to be detected across long evolutionary distances.

**Table 3:** Candidates based on systemic RNAi genes found in *C. elegans*

Gene name	Ce gene ID	Tc gene ID	Biological function	Reference
*Sid-1*	CO4F5.1	11760	Systemic RNAi (somatic cells)	[53]
		06161		
		15033		
*Rsd-2*	F52G2.2		Systemic RNAi (germ cells)	[54]
*Rsd-3*	C34E11.1	12168	Systemic RNAi (germ cells)	[54]
Epn-1*	T04C10.2	05393	Endocytic protein (EPsiN)	
*Rsd-6*	F16D3.2		Systemic RNAi (germ cells)	[54]

* Related to *Rsd-3*. Ce: *Caenorhabditis elegans*. Tc: *Tribolium castaneum*.

The third gene, *rsd-3*, encodes a protein that contains an epsin amino-terminal homology (ENTH) domain [60]. ENTH domains are often found in proteins involved in vesicle trafficking, suggesting the possible involvement of endocytosis in systemic RNAi [60]. We found a homolog for Rsd-3 in *Tribolium *(Tc-Rsd3). *Drosophila *also carries a protein similar to Rsd-3 (Epsin-like).

In addition, the Rsd-3 protein has a close relative in *C. elegans*, Epn-1, whose *Drosophila *counterpart (Liquid Facets; Lqf) has been reported to be involved in Notch signaling [96-98]. We found a *Tribolium *ortholog for Epn-1/Lqf, which we named Tc-Lqf. Although there is no report implying the involvement of Epn-1/Lqf family proteins in systemic RNAi, their high degree of identity with Rsd-3 proteins suggest that such a role is possible.

Since *Drosophila *(which seems to lack a systemic RNAi response) also carries Rsd-3-like proteins (Table 3), it does not seem likely that these proteins determine the presence or absence of systemic RNAi in insects. However, it might be still possible that the expression level and/or tissue specificity of *rsd-3*-like genes affect the degree of RNAi efficiency.

#### Endocytosis components and scavenger receptors

Another piece of evidence that suggests the involvement of endocytosis in dsRNA uptake comes from a study using *Drosophila *S2 culture cells [61,62]. Among the factors identified in this study as necessary for dsRNA uptake are a number of proteins whose functions are implicated in endocytosis [61,62] (Table 4). Also, several scavenger receptors, such as Eater and Sr-CI, were found to be important for dsRNA uptake [61,62] (Table 4). Scavenger receptors are known to act as receptors for large molecules and/or microbes (for a review of scavenger receptors, see [99]). Since these receptors act in phagocytosis, a type of endocytosis, they could potentially act as receptors for dsRNA molecules in an endocytic process. Although the factors identified in S2 cells might reflect mechanisms of dsRNA uptake specific to hemocyte-like cells [100], it is possible that some of them function in other tissues as well.

**Table 4:** Candidate genes for dsRNA pptake in *T. castaneum*

Gene name	Dm gene ID	Tc gene ID	Biological function	Reference
*Arf72A*	CG6025	08443	Endosome transport	[62]
*AP 50*	CG7057	11923	Endocytosis	[62]
*Clathrin hc*	CG9012	15014	Endocytosis	[62]
*IdICP*	CG6177	10886	Exocytosis	[62]
*Light*	CG18028	15204	Lysosomal transport	[62]
*Nina C*	CG54125	14087	Rhodopsin mediated signaling	[62]
*Rab 7*	CG5915	06036	Endosome transport	[62]
*Eater*	CG6124	XP_969372*	Inate immune response/phagocytosis	[61]
*Sr-CI*	CG4099		Inate immune response/phagocytosis	[61]
*Sr-CII*	CG8856	15640	Inate immune response/phagocytosis	[61]
*Sr-CIII*	CG31962		Inate immune response/phagocytosis	[61]
*Sr-CIV*	CG3212		Inate immune response/phagocytosis	[61]
*Vha16*	CG3161	11025	ATP synthase/ATPase	[62]
*VhaSFD*	CG17332	06281	ATP synthase/ATPase	[62]
*Gmer*	CG3495	14956	Metabolism	[62]
*P13K59F*	CG5373	00620	Lipid metabolism	[62]
*Saposin r*	CG12070	00449	Lipid metabolism	[62]
*Egghead*	CG9659	08154	Oogenesis	[62]
	CG4572	02692	Peptidase	[62]
	CG5053	07768	Signal transduction	[62]
	CG8184	04152	Ubiquitin ligase	[62]
	CG8773	16254	Peptidase	[62]
	CG5382	09067	Zinc finger transcription factor	[62]
	CG5434	12172	Translation regulation	[62]
	CG3248	12410	Unknown	[62]
	CG3911	14009	Unknown	[62]
	CG8671	04825	Unknown	[62]
	CG5161	07973	Unknown	[62]

*XP_969372 is a NCBI prediction that partially matches Tc_02053; however, Tc_02053 seems to be a chimera of at least three genes. Dm: *Drosophila melanogasater*. Tc: *Tribolium castaneum*.

We have identified the *Tribolium *orthologs of these genes (Table 4). In many cases, they show clear one-to-one orthology. In the case of *Sr-CI*, however, we found only one *Tribolium *homolog (*Tc-Sr-C*), in contrast to four closely related paralogs in *Drosophila *[100]. The case of *eater *is even more complicated. *eater *encodes a Nimrod family protein that contains multiple NIM-type EGF domains [101,102]. A BLAST search using *Drosophila *Eater (CG6124) identifies several predicted *Tribolium *proteins that contain NIM repeats. Among them, an NCBI predicted protein (XP_969372) and Eater are reciprocal best hits, suggesting that they might be orthologous. However, the similarities among Nimrod family proteins in both insects make it difficult to assign orthology. Detailed sequence analysis will be required to definitively determine the orthology of these genes.

As in the case of *rsd-3*, the fact that both *Drosophila *and *Tribolium *carry these genes might suggest that these factors do not determine the presence or absence of systemic RNAi in insects. Yet, it is still possible that a difference in tissue specificity and/or expression level might affect the efficiency of dsRNA uptake from the outside environment. Expression analysis of these genes might help determine whether these factors are broadly involved in dsRNA uptake in insects.

### Functional analysis of Dicer, Argonaute, and Sil genes in *Tribolium*

**Figure 7:**
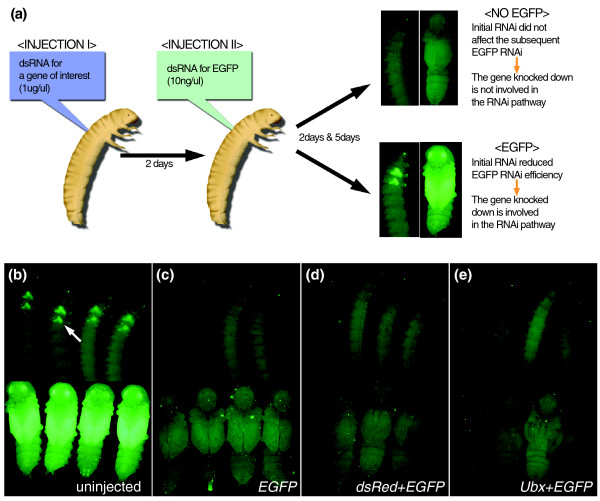
An in *vivo *assay system for RNAi genes in *Tribolium*. **(a) **A scheme of the *in vivo *assay system for RNAi genes. **(b) **Uninjected Pu11 larvae and pupae. EGFP is expressed in the wing primordia (arrow) at the larval stage, as well as in the pupal wings. EGFP is also expressed in the eye. **(c) **EGFP dsRNA-injected larvae and pupae. EGFP expression is completely silenced. **(d, e) **Larvae and pupae that were injected with dsRNA for *dsRed *(d) or *Tc-Ubx *(e) prior to the secondary *EGFP *RNAi. The prior injection of dsRNA for these genes does not affect the effectiveness of *EGFP *RNAi. *Tc-Ubx *RNAi pupae show hindwing to elytron transformation (e), indicating that the RNAi is working properly.

To further understand the RNAi mechanism in *Tribolium*, we have developed an assay system to assess the involvement of genes in the RNAi pathway *in vivo*. This system takes advantage of an enhancer trap line (Pu11), which expresses enhanced green fluorescence protein (EGFP) in the eyes and in the future wing primordia (Figure 7a, b). We previously reported that the RNAi effect can be monitored by the intensity of EGFP fluorescence in the Pu11 line after *EGFP *RNAi [27]. Our assay system is composed of three steps (Figure 7a): first, RNAi for a gene of interest at the early last larval stage, prior to the onset of wing EGFP expression in Pu11; second, RNAi for *EGFP *two days after the initial injection; and third, monitoring of the EGFP expression at the prepupal and mid-pupal stages when Pu11 EGFP expression is most intensified. In this assay system, the efficiency of *EGFP *RNAi would be reduced (therefore, EGFP expression would be visible) if the gene knocked down at the first step is involved in the RNAi pathway. In contrast, EGFP expression would be silenced if the initially knocked down gene is not involved in the RNAi pathway. In order to maximize the sensitivity of this test, the initial RNAi is done at a high concentration (1 μg/μl, approximately 0.5 μg/larva) while the second RNAi for *EFGP *is performed at a lower concentration (10 ng/μl, approximately 5 ng/larva). This low concentration of *EGFP *dsRNA is enough to completely silence EGFP expression (*n *= 147; Figure 7c) when injected alone. A potential caveat of this system is that injection of the initial dsRNA might saturate the RNAi machinery and prevent the effect of the secondary RNAi in a non-specific manner. In this case, a low concentration of the secondary dsRNA might exacerbate the problem.

To rule out such a problem, we first tested our assay system with genes that are not involved in the RNAi pathway. dsRNA for *dsRed *(an exogenous gene) was injected prior to *EGFP *RNAi, which resulted in complete silencing of EGFP (*n *= 27; Figure 7d). We also used *Tc-Ultrabithorax *(*Tc-Ubx*) as a control for an endogenous gene. *Tc-Ubx *RNAi induced a hindwing to elytron transformation as described before [103], but did not affect the EGFP silencing (Figure 7e). The prior injection of dsRNA for either an exogenous or an endogenous gene did not affect the effectiveness of *EGFP *RNAi, indicating that our assay system does not have a competition problem.

**Figure 8:**
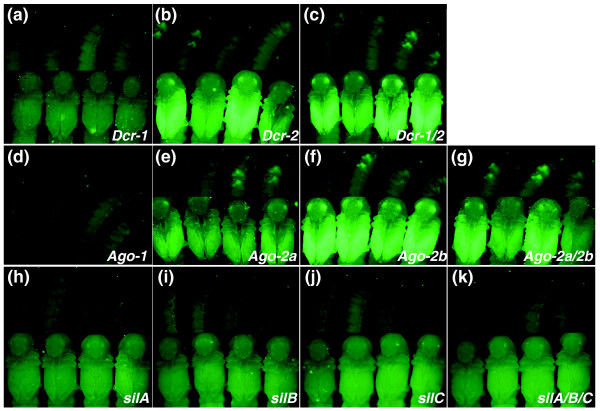
Functional analysis of Dicer, Argonaute, and *sil *genes in *Tribolium*. Larvae and pupae that were injected with dsRNA for **(a-c) **Dicer, **(d-g) **Argonaute, or **(h-k) ***sil *genes prior to EGFP RNAi. RNAi for *Dcr-2*, *Ago-2a*, or *Ago-2b *reduces the efficiency of *EGFP *RNAi (b, c, e-g). In contrast, RNAi for *Dcr-1*, *Ago-1*, or *sil *genes does not affect *EGFP *RNAi (a, d, h-k).

One of the findings through our genome-wide survey for RNAi genes in the *Tribolium *genome is that *Tribolium *has more RNAi component genes than *Drosophila*, which might make *Tribolium *more sensitive to dsRNA. We have cloned the *Tribolium *Argonaute and Dicer genes and tested whether these genes are actually involved in the RNAi pathway. We found that RNAi for *Dcr-2 *decreases the effectiveness of subsequent *EGFP *RNAi (Figure 8b; 10 of 17 individuals show EGFP expression), indicating that *Dcr-2 *is important for the RNAi pathway. However, contrary to our analysis of the Dcr-1 protein domain architecture (which suggests the possible involvement of *Dcr-1 *in the RNAi pathway), RNAi for *Dcr-1 *had no effect on *EGFP *RNAi (*n *= 32; Figure 8a), and did not enhance the effect of the *Dcr-2 *RNAi in the *Dcr-1/2 *double RNAi (Figure 8c; 12 of 30 show EGFP expression). Instead, Dcr-1 RNAi shows an occasional wing expansion defect (3 of 20), suggesting that *Dcr-1 *is involved in wing development, most likely through the miRNA pathway.

Of the three *Tribolium *Argonaute genes, we found that both *Ago-2 *genes are involved in the RNAi pathway (Figure 8e-g; 8 of 28 *Ago-2a *RNAi and 12 of 28 *Ago-2b *individuals show EGFP expression). This is in line with our hypothesis, and indicates that *Tribolium *indeed has duplicated Argonaute genes that are functional in the RNAi pathway. Larvae injected with *Ago-1 *dsRNA show developmental defects and fail to pupate, but still exhibit efficient *EGFP *silencing (Figure 8d; *n *= 21). This result suggests that *Ago-1 *is involved in the miRNA pathway, but not in the RNAi pathway.

We also tested whether the *Tribolium sil *genes are involved in the RNAi pathway. Neither the single RNAi for each *sil *gene nor the triple RNAi shows any effect on subsequent *EGFP *RNAi (Figure 8h-k), suggesting that the *sil *genes are not involved in systemic RNAi in *Tribolium*. This result is consistent with our *tag-130 *deletion mutant analysis in *C. elegans*. However, this result must be interpreted with caution since triple RNAi might weaken the RNAi effect on the *sil *genes (see Discussion).

## Discussion

RNAi techniques have had tremendous impact on many biological fields. In many organisms, RNAi allows loss-of-function phenotypes to be analyzed in the absence of mutants. In some organisms such as *Tribolium*, simple injection of dsRNA into the larval or pupal body cavity can induce the RNAi response systemically [27,41]. However, some organisms (such as many lepidopteran [46]) lack the ability to respond to dsRNA systemically. Understanding the molecular basis of systemic RNAi might help us apply systemic RNAi-based methods to these insects.

*Tribolium*, which is a highly established genetic model system, has a robust systemic response to dsRNA, giving us an opportunity to explore the molecular mechanism for systemic RNAi in an animal other than *C. elegans*. In this study, we have surveyed the *Tribolium *genome for the genes that encode RNAi core components, as well as the genes that have been implicated in systemic RNAi. If the mechanism for systemic RNAi is conserved between *C. elegans *and insects, we would expect to find a component that is present in *C. elegans *and *Tribolium *but not in *Drosophila*. However, we find a surprisingly low degree of conservation between the *C. elegans *and *Tribolium *gene inventories.

In the following section, we discuss our results in the context of three steps that might be important for systemic RNAi: the cellular uptake of dsRNA; the amplification and maintenance of dsRNA; and an efficient RNAi response.

### The dsRNA uptake mechanism is not highly conserved

For a systemic response, cells must first take up dsRNA from their environment. Several proteins responsible for dsRNA uptake have been discovered in *C. elegans*. The best described is Sid-1, which can confer the ability to import dsRNA to *Drosophila *cells in a cell culture environment [59]. The finding of three *sid-1 *homologs in *Tribolium *but none in *Drosophila *appears on the surface to be a convincing explanation for the ostensible lack of systemic RNAi in *Drosophila*.

We challenge this assumption with two lines of evidence. The first evidence comes from the fact that all *sid-1 *homologs in *Tribolium *(and other organisms) have more identity with another *C. elegans *gene, *tag-130*, than with *sid-1*. Importantly, these proteins share several blocks of identity in the extracellular amino-terminal domain that are not present in *C elegans *Sid-1. Since the extracellular domain is likely important for ligand specificity, this conservation suggests that the function of Sil proteins in *Tribolium *might be more similar to that of Tag-130 than Sid-1. Further, we have shown that the *tag-130 *gene is not required for systemic RNAi in *C. elegans*. These data raise the possibility that the dsRNA uptake function of *sid-1 *has evolved in a nematode lineage, and is not an ancestral feature of *tag-130 *homologs. *C. elegans *is known to exhibit an exceptionally high rate of amino acid change [104]. The long branch of *C. elegans *Sid-1 in the phylogenetic tree might support the idea that Sid-1 has diverged quickly, and gained a function that is not conserved in other organisms.

The second line of evidence comes from the apparent breakdown in the correlation between systemic RNAi and *sil *genes (Table 1). We note that the silkworm moth, *B. mori*, has similar *sil *genes but efforts to apply systemic RNAi on this species have been unsuccessful (S Tomita, unpublished data; R Futahashi and T Kusakabe, personal communications; but also see [47-49] for some successes). In contrast, some tissues in adult dipterans have been shown to be capable of taking up dsRNA [33-37,105], although these insects lack *sid-1*-like genes. Parental RNAi has also been performed successfully in *Drosophila *[45,106]. In addition, the parasitic nematode species *Haemonchus contortus *shows an ability to respond to soaking RNAi [107], but *sid-1/tag-130 *related genes can not be found in its sequenced genome [107] (data not shown).

Taken together, these observations suggest that a Sid-1-based mechanism is not the only existing method of dsRNA uptake. Triple RNAi for the three *sil *genes does not affect subsequent RNAi for EGFP (Figure 8h-k), suggesting that *sil *genes are dispensable for systemic RNAi in *Tribolium*. However, the use of multiple RNAi complicates interpretation of this result. Competition for RNAi components by multiple dsRNA triggers is known to weaken the efficiency of RNAi in *C. elegans *[83] as well as in *Tribolium *(SCM and YT, unpublished data). Thus, it is still possible that *sil *genes are required for systemic RNAi, but that the competition produced by triple RNAi results in incomplete knockdown of the *sil *genes. The stability of Sil proteins could also affect our assay. Some amount of the Sil proteins might stay functional even two days after knockdown of the *sil *genes. In these cases, the systemic RNAi pathway would retain some function. While the above concerns need to be considered, we do see a delay in larval development in the triple *sil *RNAi (data not shown), but not in each single RNAi. This might suggest that the triple RNAi is efficiently removing *sil *gene function. Further functional analysis, such as attempts to rescue *C. elegans sid-1 *mutants with *Tribolium sil *genes, or overexpression of *sil *genes in *Drosophila *culture cells, might reveal whether insect Sil proteins are capable of promoting dsRNA uptake.

Three more genes, *rsd-2, rsd-3*, and *rsd-6*, have been identified as important factors for the systemic RNAi response in germline cells in *C. elegans *[60]. Two of them, *rsd-2 *and *rsd-6*, are either not present in the *Tribolium *genome or their sequence is rapidly evolving such that homology cannot be detected over long evolutionary distances. Only *rsd-3 *has a clear ortholog in *Tribolium *(*Tc-epsin-like*). However, *Drosophila *also has an *epsin-like *gene but does not show clear systemic RNAi. Either the gene is not sufficient to confer enough dsRNA uptake for systemic RNAi or the expression of this gene is more restricted in *Drosophila*.

In summary, the genes required for systemic RNAi in *C. elegans *are not highly conserved in *Tribolium*. These findings contradict the recent suggestion that the presence of *sid-1 *like genes is sufficient for a systemic RNAi response in an organism [28,53,93]. A different mechanism for dsRNA uptake, such as an endocytosis-based mechanism, might await discovery in *Tribolium*.

### The *C. elegans *RNAi amplification mechanism is not present in *Tribolium*

In *C. elegans *(as well as in plants and fungi), RdRP amplifies the RNAi effect [19,20]. This amplification is apparently absolutely necessary for the RNAi response in *C. elegans*, as RdRP mutants are insensitive to dsRNA [19,20]. The robust RNAi response in *Tribolium *might be due to an amplification mechanism similar to that in *C. elegans*. However, we do not find RdRP in *Tribolium*. Although we cannot exclude the possibility that an RdRP gene is located in the non-sequenced part of the *Tribolium *genome, this possibility is unlikely for several reasons. First, RdRP genes do not seem to be present in most animals (see the 'Absence of RNA-dependent RNA polymerase in *Tribolium*' section). Second, RdRP-amplified siRNAs are associated with a specific class of Argonautes (secondary Argonautes) in the two-step RNAi mechanism of *C. elegans*, but secondary Argonautes are not conserved in *Tribolium*. And third, in *Tribolium*, it is possible to target a particular isoform of a gene by RNAi, even when it shares more upstream sequence with other isoforms (lack of transitive RNAi) [108]. This indicates that there is no RdRP activity in *Tribolium*, since such activity would result in production of siRNAs corresponding to the region upstream (and perhaps even downstream) of the initial dsRNA target site and the knockdown of all messages sharing the upstream sequence. If there is an RNAi amplification step in *Tribolium*, it must be based on a different mechanism.

### The *Tribolium *RNAi machinery could be more efficient than that of *Drosophila*

The inventories of genes involved in systemic RNAi and amplification do not show clear differences between *Tribolium *and *Drosophila *that would explain the presence and absence of systemic RNAi. An alternative hypothesis is that the core machineries for RNAi might function with different efficiencies in these species. Our genomic survey for RNAi core components has indeed revealed several differences in the number of these core component genes between *Tribolium *and *Drosophila*. *Drosophila *carries only one *Ago-2 *gene, while the *Tribolium *genome has two, apparently due to a lineage-specific duplication. We have shown that both *Tribolium Ago-2 *genes are involved in the RNAi pathway (Figure 8d-g). As the availability of Ago proteins has been shown to determine the RNAi efficiency [80], it is quite possible that the *Ago-2 *copy number allows a more efficient RNAi response in *Tribolium *than in *Drosophila*.

There are also interesting differences between Dicer proteins. Dicer proteins can be involved in both RNAi and miRNA pathways [7]. For example, Dcr-1 protein in *C. elegans *functions in both pathways [67,69]. In *Drosophila*, however, a subfunctionalization seems to have happened between the two Dicer genes; one Dicer (Dcr-1) works specifically in the miRNA pathway, while the other (Dcr-2) functions in the RNAi pathway [17]. Each of these Dicer proteins has unique domain losses [17] (Figure 1c). One of the *Tribolium *Dicer proteins, Tc-Dcr-2, has similar domain architecture to *Drosophila *Dcr-2, suggesting that Tc-Dcr-2 is involved in the RNAi pathway. In contrast, Tc-Dcr-1 has an amino-terminal helicase domain, which is lacking in Dm-Dcr-1. This makes the domain architecture of Tc-Dcr-1 more similar to *C. elegans *Dcr-1, which might suggest that, in addition to Dcr-2, Dcr-1 could also be involved in the RNAi pathway in *Tribolium*. Our assay system provided no evidence that *Dcr-1 *is also involved in the RNAi pathway (Figure 8a). However, Dcr-1 RNAi does produce developmental defects. This could be due to defects in the miRNA pathway, since miRNAs play a crucial role in development in *Drosophila *and other organisms [109]. We noticed that the *Dcr-1 *RNAi phenotype is weaker than that of *Ago-1*, which is also likely to be involved in the miRNA pathway. This difference suggests that there might be another factor that acts redundantly with Dcr-1. This redundancy might also influence the Dcr-1 function in the RNAi pathway, leaving open the possibility that *Dcr-1 *is involved in the RNAi pathway but that its RNAi effect is masked in our assay system by a redundant factor. Dcr-2 is not the redundant factor since the *Dcr-1/2 *double RNAi phenotype is still weaker than that of *Ago-1*. Further functional analysis is necessary to unravel the involvement of Dicer genes in the RNAi pathway in *Tribolium*.

In line with this, the *Tribolium *genome contains an additional R2D2-like dsRBM gene compared to *Drosophila*. *R2D2 *determines the specificity of Dicers in *Drosophila*; Dcr-1 can associate with siRNA in *R2D2 *mutants [11,14]. An additional R2D2 in *Tribolium*, with *C. elegans*-type Tc-Dcr-1, might contribute to the robust RNAi response in *Tribolium*. Alternatively, since dsRBM proteins are known to bind to dsRNA, they might be involved in the maintenance of dsRNA in cells. In that case, the presence of an additional dsRBM protein might allow a longer-lasting RNAi effect. Intriguingly, it has been suggested that an ATP-dependent mechanism might be involved in retaining dsRNA inside cells in *C. elegans *[59], implying the existence of a yet to be found dsRNA maintenance mechanism.

These differences in core RNAi components might allow *Tribolium *cells to respond to dsRNA more sensitively than *Drosophila *cells. Further functional analysis is necessary to understand the molecular mechanism underlying the systemic RNAi response in *Tribolium*, as well as the evolutionary changes that caused the difference in ability of *Tribolium *and *Drosophila *to respond to dsRNAsystemically.

### Ancestral gene set for RNAi machinery

Our genome-wide survey for RNAi genes has revealed that the repertoire of RNAi genes has been diversified even among insect species. Although the comparison between *Tribolium*, *Drosophila*, and *C. elegans *has clearly illuminated diversity in the inventory of RNAi component genes (Additional data file 8), more species will be necessary for the reconstruction of an ancestral RNAi gene set. The RNAi pathway is conserved not only in animals but also among many eukaryotes such as fungi, plants, and protists [2,110,111]. Phylogenetic analysis including diverse species might shed light on understanding the ancestral gene set and evolution of RNAi machinery.

## Conclusion

Our analysis does not find a highly conserved mechanism for systemic RNAi between *C. elegans *and *Tribolium*. Insect systemic RNAi is likely, therefore, to be based on a different mechanism that remains to be uncovered. Understanding this process would assist with rendering other insects amenable to systemic RNAi, which in many cases is a prerequisite for functional gene analysis. In addition, knowing the mechanism of systemic RNAi in insects is likely to influence approaches of pest control in which dsRNAs are produced by a host plant. With its robust systemic RNAi response [27,41], recently sequenced genome [66] and available genetic tools [112-119], *Tribolium *offers an excellent opportunity to uncover the molecular basis of systemic RNAi in insects.

## Materials and methods

### Manual curation of automatically annotated *Tribolium *genes

*Tribolium *homologs were identified by BLAST search at BeetleBase [120], and the corresponding predicted protein sequences were obtained from the *Tribolium castaneum *Genome Project website at the Baylor College of Medicine website [121]. These sequences were used for dot-plot analyses [122] and ClustalW alignments with *Drosophila *and other orthologs. Predicted exons that showed no identity to other orthologs were deleted. Conversely, when the *Tribolium *predictions were missing regions conserved in other orthologs, we searched the appropriate region of *Tribolium *genomic sequence for exons missed by the predictions.

### Phylogenetic analysis

Multiple alignments were created and curated in MEGA 3.1 (Additional data file 1) [123]. Neighbour-joining analysis was performed in MEGA 3.1 with bootstrapping using 1,500 or 5,000 replicates. The same alignments were also used in TreePuzzle for maximum likelihood analysis [123,124] using standard settings. The trees were visualized using TreeView [125]. Both types of analysis resulted in essentially the same relationships.

The following conserved domains were used to create multiple alignments: Piwi domain for Argonaute proteins; first RNaseI domain for the Dicer protein alignment including Drosha; full-length except for dsRBM for the Dicer protein alignment without Drosha; tandem dsRBM for R2D2/Loquacious/Pasha; RdRP domain for RdRP proteins; exonuclease domain for Eri-1-like exonuclease; and multiple transmembrane domain (corresponds to TM2-TM11 portion of *C. elegans *Sid-1) for Sid-1-like proteins (Additional data file 1).

### Search for RdRP orthologs

*S. pombe *RdP1 and *C. elegans *Ego proteins were used as the query in a tBLASTn search of the NCBI database (first searching all organisms and subsequently restricted to Eukaryota and then to Metazoa) to identify RdRP homologs. Sequenced nematode genomes were also searched at Nematode.net [126]. Viral RdRPs were excluded from the analysis, since they were not identified by these searches and ClustalW did not produce reasonable alignments of eukaryotic and viral RdRPs.

### Domain analysis

Domain architecture of Dicer proteins was analyzed by ScanProsite [72,.127]. The scores for each protein domain presented in Figure 1c are similarity scores produced by a PROSITE search. A query sequence is compared to the PROSITE protein domain database. Domains are represented as a 'profile', which is a table of position-specific amino acid weights and gap costs. These numbers are used to calculate a similarity score for any alignment between a profile and a sequence. An alignment with a similarity score higher than or equal to a given cut-off value indicates a motif occurrence. Similarity scores below 8.5 are typically (but not for all profiles) regarded as questionable. See details at the PROSITE website [128]. Sid-1-like proteins were analyzed by TMHMM server v2.0 [129] and InterProScan [130].

### Cloning genes

Total RNA was isolated from *Tribolium *pupae (or adult *Drosophila *in the case of Dm-Ago3) using the RNeasy Protect Mini Kit (Qiagen, Valencia, CA, US), and cDNA was synthesized with SuperScript III (Invitrogen, Carlsbad, CA, USA) using oligo dT primer. Primers for *Tc-sil *genes and *Tc-snp *were designed based on the conserved domains identified by BLASTx analysis using Ce-Sid-1 and Ce-Eri-1, respectively. Primers for Argonaute and Dicer genes were designed based on conserved domains identified by BLASTx analysis using *Drosophila *homologs. Subsequent RACE analysis was performed using the GeneRacer kit (Invitrogen) on mRNA isolated from *Tribolium *pupae by the QuickPrep micro mRNA Purification Kit (GE Healthcare Bioscience, Piscataway, NJ, USA).

### Isolation of *Bombyx sil *genes

Silkworm whole genome shotgun contigs and scaffolds at KAIKObase [131] were searched with tBLASTn using *Tc-sil *genes as queries. Three scaffolds, 000945, 000542 and 002595, were revealed to contain conserved domains. Primers were designed based on the sequences of these scaffolds and partial cDNAs were amplified by RT-PCR using mRNA isolated from *Bombyx *eggs at 72-120 hours after oviposition. Full-length cDNA clones were isolated and sequenced by screening cDNA libraries made from eggs 40 hours after oviposion [132] and midgut of the fifth instar day-2 larva (gift from Dr Kazuei Mita, National Institute of Agrobiological Sciences, Japan) with the partial cDNAs as probes.

### Semi-quantitative RT-PCR

cDNA was synthesized with SuperScript III (Invitrogen) using mRNA isolated from five developmental stages (0-72 hour embryos, third/fourth instar larvae, last instar larvae, pupae, and adults). PCR was performed using Ex-Taq polymerase (Takara Bio USA, Madison, WI, USA). Reactions were performed with a varying number of cycles to ensure that comparisons were made within the linear range.

### *C. elegans *mutant strains and feeding RNAi

Two *tag-130 *alleles, *tag-130*^*gk*245 ^(strain name: VC452, created by the *C. elegans *Reverse Genetics Core Facility at UBC) and *tag-130*^*OK*1073 ^(RB1095, created by the *C. elegans *Gene Knockout Project at OMRF) were obtained from the *Caenorhabditis *Genetic Center at the University of Minnesota. The *sid-1*^*sq*2 ^strain and *unc-22 *feeding RNAi *Escherichia coli *strain were the kind gift of Dr K Morita and Dr M Han (University of Colorado at Boulder, USA). Feeding RNAi was performed as previously described by Kamath *et al*. [133], and the *unc-22 *twitching RNAi phenotype was scored under a stereomicroscope. Four independent replicates of the feeding experiment were performed for each genotype.

### *tag-130 *mutant lesion

Genomic DNA was isolated from *tag-130 *mutants (as well as N2 and *sid-1*^*sq*2 ^strains) by adapting a protocol for isolation of genomic DNA from a single *Drosophila *adult [134]. To determine the deleted region, five sets of primers that together cover the entire *tag-130 *gene were used to survey the *tag-130 *locus. We used [gk245iF1:gtgcatcgtatgagcctgtg]/[gk245iR1:aattgttgcagacgtggtca] and [OK1073D F1:ctaggtgcaatcagtgagccagtg]/[OK1073E R1:ataaaattccggcacaagtccag] to amplify the genomic region that contains the deleted region in *tag-130*^*gk*245 ^and *tag-130*^*OK*1073^, respectively. PCR products were then cloned into pCR4-TOPO using the TOPO TA-Cloning Kit for Sequencing (Invitrogen), and sequenced to determine the deleted region [GenBank: EF695395, EF695396]. Total RNA was isolated from *tag-130 *mutants and the *sid-1*^*sq*2 ^and N2 strains using the RNeasy Protect Mini Kit (Qiagen), and cDNA was synthesized with SuperScript III (Invitrogen) using oligo dT primer. We used [tag130cDNA F2:aagagcgtatacacatttggaaga] and [tag130cDNA R3:attacattgatggcggtgaaa] for RT-PCR. We detected two different transcripts in *tag-130*^*OK*1073^, both of which were cloned into pCR4-TOPO and sequenced [GenBank: EF695397, EF695398].

### dsRNA synthesis and larval injection in *Tribolium*

A PCR amplified fragment of each gene was cloned into pCR4-TOPO using the TOPO TA Cloning Kit for Sequencing (Invitorogen). Primers used to amplify gene fragments and the size of each fragment are summarized in Additional data file 9. Templates for *in vitro *transcription were prepared by PCR using a primer designed to prime on two pCR4-TOPO vector regions that flank the inserted gene fragment [TOPO_RNAi_T7:taatacgactcactatagggcgaattcgccctt]. This primer amplifies a gene fragment with the T7 polymerase promoter site at both ends. Gene specific primers with the T7 sequence at their 5' end were used to create a EGFP dsRNA template (520 bp) [GFPiF2:taatacgactcactatagggcgatgccacct, GFPiR5: taatacgactcactatagggcggactgggtg] (the T7 site is underlined). dsRNA synthesis (using an Ambion MEGAscript T7 High Yield Transcription Kit. Ambion, Austin, TX, US) and larval injection were performed as described previously [27]. Larvae and pupae were documented using an Olympus SZX12 microscope with Nikon DXM 1200F digital camera. The same exposure time (1/6 second) was used for all images.

### Accession numbers and gene names

The gene names given for GLEAN genes are summarized in Additional data file 7.

#### GenBank accession numbers

*Dm-Ago3 *[GenBank:EF688531], *Tc-snp *[GenBank:EF688530], *Tc-silA *[GenBank:EF688527], *Tc-silB *[GenBank:EF688528], *Tc-silC *[GenBank:EF688529]. *Tc-Ago-1 *[GenBank:EU273915], *Tc-Ago-2a *[GenBank:EU273916], *Tc-Ago-2b *[GenBank:EU273917], *Tc-Dcr-1 *[GenBank:EU273918], *Tc-Dcr-2 *[GenBank:EU273919], *Tc-R2D2 *[GenBank:EU273920], *Tc-C3PO *[GenBank:EU273921], *tag-130 *deletion lesions [GenBank:EF695395, EF695396], *tag-130*^*OK*1073 ^isoforms [GenBank:EF695397, EF695398].

### DNA Data Bank of Japan accession numbers

*Bm-sil1 *[DDBJ:AB327183], *Bm-sil2 *[DDBJ:AB327185], *Bm-sil3 *[DDBJ:AB327184].

## Abbreviations

dsRBM, dsRNA binding motif; dsRNA, double-stranded RNA; EGFP, enhanced green fluorescence protein; miRNA, micro-RNA; miRNP, micro-RNA ribonucleoparticle; RdRP, RNA-dependent RNA polymerase; RISC, RITS, RNA-induced initiation of transcriptional gene silencing; RNA-induced silencing complex; RNAi, RNA interference; siRNA, short interfering RNA.

## Authors' contributions

YT and GB conceived and designed the experiments. GB and DG performed the annotations of the Dicer, Argonaute, and RdRP proteins. YT and SCM performed annotations of Dicer, dsRBM, Eri-1, Sid-1-related proteins, and candidate factors for systemic RNAi. YT performed the *Drosophila *gene cloning (*Dm-Ago-3*) and *C. elegans *experiments. YT and SCM performed *Tribolium *gene cloning and RNAi experiments. ST cloned the *sid-1*-like genes of *Bombyx*. MS contributed the initial annotation of *sid-1*-related genes. YT, SCM, and GB wrote the paper. All authors discussed the results and commented on the manuscript.

## Additional data files

The following additional data are available with the online version of this paper. Additional data file 1 contains multiple alignments used for pylogenetic analyses. Additional data file 2 is a phylogenetic tree for Eri-1-like nucleases including *C. elegans *Crn-4. Additional data file 3 is *sil *gene expression profile in *Tribolium*. Additional data file 4 is a multiple alignment of full-length Sil proteins. Additional data file 5 contains Dot-matcher alignments of Sil proteins. Additional data file 6 shows *C. elegans tag-130 *locus and deletions. Additional data file 7 is a list of GLEAN gene number and corresponding gene names. Additional data file 8 is a table showing RNAi and miRNA components in *Tribolium, Drosophila *and *C. elegans*. Additional data file 9 is a table showing primers used for dsRNA synthesis.

## Supplementary Material

Additional data file 1 Multiple alignments used for pylogenetic analyses. Click here for file

Additional data file 2 Phylogenetic tree for Eri-1-like nucleases including *C. elegans *Crn-4. Click here for file

Additional data file 3 *sil *gene expression profile in *Tribolium*. Click here for file

Additional data file 4 Blue boxes indicate conserved portions of the amino-terminal extracellular domain shown in Figure 6. Amino acids making up predicted transmembrane regions for each protein are shown in orange, while the 11 predicted transmembrane domains of Tc-SilA protein are denoted with orange bars. The region corresponding to TM2 to TM11 (delimited by green arrows) was used for phylogenetic analysis. Click here for file

Additional data file 5 Dot-matcher alignments of Tc-SilA protein with Sid-1-like proteins from various organisms. Conservation between two proteins is visualized as a diagonal line. Tc-SilA does not show high conservation with Ce-Sid-1 in the amino-terminal extracellular region (A, red box), but shows conservation with Ce-Tag130 (B, red box). Additional conservation is seen in the carboxy-terminal transmembrane domains (B, blue boxes), which is lacking in Ce-Sid-1(A, blue box). These conserved domains are seen in all Sid-1-like proteins examined (B-F), except Ce-Sid-1 (A). Click here for file

Additional data file 6 (A) *tag-130 *gene exon/intron structure. The regions deleted in *tag-130*^*gk*245 ^and *tag-130*^*OK*1073 ^are indicated with orange bars. (B) An enlargement of the *OK1073 *deleted region and schematic diagrams of two mRNA forms detected in *tag-130*^*OK*1073 ^mutants. The deleted region is indicated in orange. In isoform *OK1073-A*, which is the more abundant of the two forms, the remaining portion of intron 13 is not spliced out and is juxtaposed with the remaining portion of exon 17. Intron 13 contains a stop codon in this reading frame, which should cause truncation of the protein. In the other isoform (*OK1073-B*), the remaining portion of intron 13 is spliced out along with the remaining portion of exon 17 (and intron 17), juxtaposing exon 13 with exon 18. This changes the reading frame in exon 18, and should also result in premature truncation of the protein. Click here for file

Additional data file 7 GLEAN gene number and corresponding gene names. Click here for file

Additional data file 8 RNAi and miRNA components in *Tribolium, Drosophila *and *C. elegans*. Click here for file

Additional data file 9 Primers used for dsRNA synthesis. Click here for file
