# Supplementary material for: Exploring systemic RNA interference in insects: a genome-wide survey for RNAi genes in Tribolium
Source: Genome Biol. 2008 Jan 17;9(1):R10. doi: 10.1186/gb-2008-9-1-r10 (PMC2395250; doi:10.1186/gb-2008-9-1-r10)
Supplement: Additional data file 1 — Multiple alignments used for pylogenetic analyses. [file gb-2008-9-1-r10-S1.pdf]

S1. Multiple Alignments used for pylogenetic analyses

Dicer proteins (without Drosha)

```
Tc-Dicer1    1 PREYQVELLDSAKKRNTIVCSSASSAKAFITIKLLQEFSHKMRVPHG----KQALFVLDG
Dm-Dicer1    1 PRDFQVELLATAYERNTIICLGHRSSKEFIALKLLQELSRRARRHGR----VSVYLSCEV
Ce-Dicer1    1 PRDYQVELLDKATKKNTIVQLGTGSGKTFIAVLLLKEYGVQLFAPLDQGGKRAFFVVEKV
Tc-Dicer2    1 PRNYQVNLMEIAIRENTIIYLPTGSGKTFIAIMVLKQLCAPILRPYSDGGKISVILVNSV
Dm-Dicer2    1 PRGYQLRLVDHLTKSNGIVYLPTGSGKTFVAILVLKRFSQDFDKPIESGGKRALFMCNTV

Tc-Dicer1   57 PNVPIMTSHVKLLTDLTVTSIDKEESLKASNVIVTTAEVCVLLCKKNFVHLDSYALIVID
Dm-Dicer1   57 GTSTEPCSIYTMLTHLTDLRVWQEQCWTDYHVSILRPEGFLYLLETRELLLSSVELIVLE
Ce-Dicer1   61 NLVEQQAIHIEVHTSFKVGQVHGQTFMKRHHVVVITAQCLLDLIRHAYLKIEDMCVLIFD
Tc-Dicer2   61 ALVDQHGKYVRDHATFSVGTYTGEMQFNKYQVVIMTSQIMVNLINNRFIDLGKVNLMIFD
Dm-Dicer2   61 ELARQQAMAVRRCTNFKVGFYVGEQEIKKNQVLVGTAQVFLDMVTQTYVALSSLSVVIID

Tc-Dicer1  117 CLYGGQ-QSLVREIMARYQAIQAPRPRILGLTAGLLGSEMQPDRLEAELQRLEKLLSSSV
Dm-Dicer1  117 DCHDSA-VYQRIRPLFENHIMPAPRPRILGLAGPLHSAGCELQQLSAMLATLEQSVLCQI
Ce-Dicer1  121 ECHHALGSQHPYRSIMVDYKLLKKVPRVLGLTASLIKAKVAPEKLMEQLKKLESAMDSVI
Tc-Dicer2  121 ECHHGVEDQPMRQIMKHFHSCTDK-PRVLGLTATLLNGNCKLSKVMDEIRSLEVTFHSKV
Dm-Dicer2  121 ECHHGTGHHPFREFMRLFTIANQTLPRVVGLTGVLIKGNE-ITNVATKLKELEITYRGTV

Tc-Dicer1  176 DTSSEILTLIRLSCRPRERIDPKEQPLSFFDDFLEILDDLGPWSADRAAYGMLIKIEKLK
Dm-Dicer1  176 ETASDIVTVLRYCSRPHEYIDPKVDPLNVINSLLVVLHEMGPWCTQRAAHHFYQCNEKLK
Ce-Dicer1  181 ETASDLVSLSKYGAKPYEVVDPRRPIKDSLKTTRAVFRQLGPWAAWRTAQVWEKELKIIK
Tc-Dicer2  180 ATVEGLDVVVGYSTNPQELFDVLKSLRNLISDLMIHIEMLGAFGGHIACVAHMIQIERIK
Dm-Dicer2  180 SDTKEMENVMLYATKPTEVM-KKSFVKQLFNDFLYQMKEYGIYAASIAIISLIVEFDIKR

Tc-Dicer1  236 VKVPYERHYLLLCVASSVLVSIRALCELEFQDYVFRFSTPKVLRFLQVLKQFKPLCALVF
Dm-Dicer1  236 VKTPHERHYLLYCLVSTALIQLYSLCEHAFHRHIERYSSPKVRRLLQTLRCFKPLCALIY
Ce-Dicer1  241 SQVLPDKTLRFLNMAKTSMITIKRLLEPEMKKILRPYVPQRVIRLFEILETFNPLSAIIF
Tc-Dicer2  240 KHCQNHQLFIVLNYVMTIMGTTKLLLEETMAGYIRKFSSDKVLKVFEILDEYKTLCCLVF
Dm-Dicer2  239 RQAETLSVKLMHRTALTLCEKIRHLLVQKLQDMIMNFSTPKVQRFLMSLKVSFAICCLVF

Tc-Dicer1  296 VKNRYKAEALFALLCVMSKSDEEYWWVSVSFSVNKIADPVREPREAESEHKRQEEVLRKY
Dm-Dicer1  296 CNQNHTARVLFELLAEISRRDPDLKFLRCQYTTDRVADPTTEPKEAELEHRRQEEVLKRF
Ce-Dicer1  301 VDQRYIAYSLLLMMRHIKSWEPKFKFVNPDYVVG-ASGRNLASSDSQGLHKRQTEVLRRF
Tc-Dicer2  300 TKRRFTAKVLHHIIDKASQVDPKFYHIKSNFVVGNKNNPYNDTRENLYITKKNREVLNSF
Dm-Dicer2  299 VERRYTCKCIYGLLLNYIQSTPELRVLTPQFMVG--RNNISPDFESVLERKWQKSAIQQF

Tc-Dicer1  356 RSHECNIMIATSALEQGCDLPKCNLVIRFDLPQSFHSYIHSKARARANEAHFLLLANNEV
Dm-Dicer1  356 RMHDCNVLIGTSVLEEGIDVPKCNLVVRWDPPTTYRSYVQCKGRARAAPAYHVILVASTT
Ce-Dicer1  360 HRNEINCLIATSVLEEGVDVKQCNLVIKFDRPLDMRSYVQSKGRARRAGSRYVITVE--E
Tc-Dicer2  360 VSKEINVLVSSNVLEEGVDIPKCTLVIKFDKSEDYRSYIQSKGRARHIKSLYYTIVETDV
Dm-Dicer2  357 RDGNANLMICSSVLEEGIDVQACNHVFILDPVKTFNMYVQSKGRARTTEAKFVLFTAKER

Tc-Dicer1  416 SDFVRYCAKLPSDTFTRLTPIWHEEKVENGSIRLPINSPVKKTVTSPPMINTLLARRAAA
Dm-Dicer1  416 KDLVKYCARLPSDTFTKLTALWRCTRNERATLRLPINSPLKHDIVGLPMPTQTLARRLAA
Ce-Dicer1  418 KDTAAYCSKLPSDIFTRLVPHNQIIPIEENELLLPINSPIKHAIVLKPMPNKKTAQMAVA
Tc-Dicer2  420 AKYDRYCSNLASDKYTTYAPEWYYE--EDSVIFLPVVCPLIDPIVGPYMHNKKDAKRAAA
Dm-Dicer2  417 EKTIRYCQTIPTDAFGFVIPWFHVLQEDERSINMPVNCMLRDTIYSDPMDNVKTAKISAA

Tc-Dicer1  476 FMICQLLHKAGELDDNLQPIGKEDPRPGTTKRRQYYYKKVADALLDCHPIIGQPTYFYKI
Dm-Dicer1  476 LQACVELHRIGELDDQLQPIGKEEPRPGTTKRRQYYYKRIASEFCDCRPVAGAPCYLYFI
```

```
Ce-Dicer1  478 LEACRQLHLEGELDDNLLPKGREAAKVGSSKRKQLYDKKIARALNESFVEADKECFIYAF
Tc-Dicer2  478 LVACIKLHQCGELDNNLLPWKKQ--DAGNKKKKRLHDKEIAPSVKSAIQPD-RVLYLHTI
Dm-Dicer2  477 FKACKVLYSLGELNERFVPKTLKTVNKADKSKDRTYKTECPLEFYDALPRVGEICYAYEI

Tc-Dicer1  536 VMKLTCPLPEEQNTRGRKIYPPEDSPQGFGILTSKEIPKISAFPIFTRSGEVSVDLQLCS
Dm-Dicer1  536 QLTLQCPIPEEQNTRGRKIYPPEDAQQGFGILTTKRIPKLSAFSIFTRSGEVKVSLELAK
Ce-Dicer1  538 ELERFREAELTLNPKRRKFEDPFNYEYCFGFLSAKEIPKIPPFPVFLRQGNMKVRLIVAP
Tc-Dicer2  535 NINPQYKRSDDLKN-AVTIYDLYKTPLKFGLLSPKPLPDLCKFPLFDSNGTLEIEIRNNV
Dm-Dicer2  537 FLEPQFESCEYTEHMYLNLQTPRN----YAILLRNKLPRLAEMPLFSNQGKLHVRVANAP

Tc-Dicer1  596 Q-LIVTENQICKIREFLNYTFTSVLRLQKYLTLFNPDASANSYLIVPTIDGAT--TTVDW
Dm-Dicer1  596 ERVILTSEQIVCINGFLNYTFTNVLRLQKFLMLFDPDSTENCVFIVPTVKAPAGGKHIDW
Ce-Dicer1  598 KKTTVTAAQLQEIQLFHNYLFTQVLQMCKTGNLEFDNAPLNTLIVPLNKRKDDMSYTINM
Tc-Dicer2  594 REVEFAANEMKEMREFHFLVFNDLLEILKEFLIFDNTGMNSEMLLVVPVQDRCGDVCVDF
Dm-Dicer2  593 LEVIIQNSQLELLHQFHGMVFRDILKIWHPFFVLDRRSKENSYLVVPLILGAGEQKCFDW

Tc-Dicer1  653 DFIDLIYANLTVLPEIIPEEVRKSYEFDPEKYRDAVVMPWYRNQDQPFYVAEICSNLNPA
Dm-Dicer1  656 QFLELIQANGNTMPRAVPDEERQAQPFDPQRFQDAVVMPWYRNQDQPFYVAEICPHLSPL
Ce-Dicer1  658 KYVSEVVANMENMPRIPKDEVRRQYKFNAEDYKDAIVMPWYRNLEQPYYVAEILPEWRPS
Tc-Dicer2  654 RVIRDNKNLKNKLEPAATERIN--LNVTEETYLHKIVSPWYRS--PPYVVTKVCPDKSAL
Dm-Dicer2  653 ELMTNFRRLPQSHGSNVQQREQQ-PAPRPEDFEGKIVTQWYANYDKPMLVTKVHRELTPL

Tc-Dicer1  713 SDFPGSDYATFEEYYLRKYSIQIQNKSQHLLDVDHTSARLNFLTPRYVNRKGVALPTSSE
Dm-Dicer1  716 SCFPGDNYRTFKHYYLVKYGLTIQNTSQPLLDVDHTSARLNFLTPRYVNRKGVALPTSSE
Ce-Dicer1  718 SKFPDTHFETFNEYFIKKYKLEIYDQNQSLLDVDFTSTRLNLLQPRIQNQPRRSRTVSNS
Tc-Dicer2  710 SRFPNHEYPNFVSYYSEKHSLSILDPSQPLLLVKGLSERLNAFKPRGAGGKRKKEKMYEE
Dm-Dicer2  712 SYMENQQDKTYYEFTMSKYGNRIGDVVHKDKFMIEVRDLTEQLTFYVHNRGKFNAKSKAK

Tc-Dicer1  773 QKQILVPELCAIHPFSASLWRKAVCLPCILYRINALLLADQIRRTVALELNLGKIELDS-
Dm-Dicer1  776 QKQILVPELCTVHPFPASLWRTAVCLPCILYRINGLLLADDIRKQVSADLGLGRQQIEDE
Ce-Dicer1  778 QRQILVPELMDIHPISATLWNVIAALPSIFYRVNQLLLTDELRETILVKAFGKEKTKLDD
Tc-Dicer2  770 LEEYLIPELVIKQEFPSCLWIQARFLPSILSRLAYLLKLQQLQVDIARGIGAKAEYLKDC
Dm-Dicer2  772 MKVILIPELCFNFNFPGDLWLKLIFLPSILNRMYFLLHAEALRKRFNTYLNLHLLPFNGT

Tc-Dicer1  832 EFKWPPLNFGWSLADVLKKSKDEEKKKQEKIEPVGPSPNVLLQALTMSNANDGINLERLE
Dm-Dicer1  836 DFEWPMLDFGWSLSEVLKKSRESKQKESLKDDTIGPSPSIILQALTMSNANDGINLERLE
Ce-Dicer1  838 NVEWNSLAYATEYEEKQTIIVKKIQQLRDLNQKSGVSPCLLLTALTTSNAADGMSLERFE
Tc-Dicer2  830 PPLELNLHLLHYEPNDPQLTQESDKSTPLIDNCLSPNLCQIYQALTAAEANDIVNLERLE
Dm-Dicer2  832 DYMPRPLEIDYSLKRNVDPLGNVIPTEDIEEPKSPAEQGEFLAAITASSAADVFDMERLE

Tc-Dicer1  892 TIGDSFLKYAITNYLYSKYENVHEGKLSHLRSKQVSNLNLYRLGRRKGLGEYMIATKFDP
Dm-Dicer1  896 TIGDSFLKYAITTYLYITYENVHEGKLSHLRSKQVANLNLYRLGRRKRLGEYMIATKFEP
Ce-Dicer1  898 TIGDSFLKFATTDYLYHTLLDQHEGKLSFARSKEVSNCNLYRLGKKLGIPQLIVANKFDA
Tc-Dicer2  890 TLGDSFLKFVASLYIIFKFPTYNEGKSTTLKGKLVSNKNLYYLGVRKNLGGILKNSDLSP
Dm-Dicer2  892 ILGDSFLKLSATLYLASKYSDWNEGTLTEVKSKLVSNRNLLFCLIDADIPKTLNTIQFTP
```

```
Tc-Dicer1  952 HDNWLPPCFYVPKELEEIPYNLVTQHSIPDKSIADCVEALIGAYLIECGPRGALLFMAWL
Dm-Dicer1  956 HDNWLPPCYYVPKELEKIPYNLVSQHSIPDKSIADCVEALIGAYLIECGPRGALLFMAWL
Ce-Dicer1  958 HDSWLPPCYIPTCDFKALPYNLLTQQHISDKSIADAVEALIGVHLLTLGPNPTLKVMNWM
Tc-Dicer2  950 SD-WVPPCFCIPQTISKNMCNFLNKQYVGDKSIADSVEALLGAYFLSGGIQGGIKFMEWI
Dm-Dicer2  952 RYTWLPPGISLPHNVLAEVNFCVGLVTIPNKVIADTLEALLGVIVKNYGLQHAFKMLEYF

Tc-Dicer1 1012 GIRVLPPREELDMLLDGYDQFERHIGYKFRDRSYLLQALTHASFSPNTLTDCYQRLEFLG
Dm-Dicer1 1016 GVRVLPATEELDQLLSGFEEFEESLGYKFRDRSYLLQAMTHASYTPNRLTDCYQRLEFLG
Ce-Dicer1 1018 GLKVIQNFLNNLWQQFQFTQLEEKIGYRFKERAYLVQAFTHASYINNRVTGCYQRLEFLG
Tc-Dicer2 1009 GILPLSTKTDVDFHMPQWREIEQRLGYTFTNRAFLLQALTHSSYSPNRITLSYERLEFLG
Dm-Dicer2 1012 KICRADNTTEIDGFLINHYYLEKNLGYTFKDRRYLLQALTHPSYPTNRITGSYQELEFIG

Tc-Dicer1 1072 DAVLDYLITRHLYEDTRMHSPGALTDLRSALVNNTIFASLAVRNGFHRYFRNLSPSLNEV
Dm-Dicer1 1076 DAVLDYLITRHLYEDPRQHSPGALTDLRSALVNNTIFASLAVRHGFHKFFRHLSPGLNDV
Ce-Dicer1 1078 DAVLDYMITRYLFEDSRQYSPGVLTDLRSALVNNTIFASLAVKFEFQKHFIAMCPGLYHM
Tc-Dicer2 1069 DAVLDFLITCYIFEHCGHLEPGQVTDLRSSLVNNNTFASLVVRCGFHKFLLMMNSNLQGH
Dm-Dicer2 1072 DAILDFLISAYIFENNTKMNPGALTDLRSALVNNTTLACICVRHRLHFFILAENAKLSEI

Tc-Dicer1 1132 VEKFVRLQEDSGHTLVDELYLVVET--EEVEDVEVPKALGDVFESVAGAIFLDSGMSLDA
Dm-Dicer1 1136 IDRFVRIQQENGHCISEEYYLLSEEECDDAEDVEVPKALGDVFESIAGAIFLDSNMSLDV
Ce-Dicer1 1138 IEKFVKLCSEDTNFNAEMYMVTTEEEIGQEEDIEVPKAMGDIFESVAGAIYLDSGRNLDT
Tc-Dicer2 1129 IDKFADYLASKNYVIDDEVLILLEEDENIAEYVDVPKVLGDIFEALAGAIYLDSNKDLKT
Dm-Dicer2 1132 ISKFVNFQESQGHRVTNYVRILLEEADNMSTNVDVPKALGDVLEALIAAVYLDCR-DLQR

Tc-Dicer1 1190 VWKVYYNMMKSEIEQFSNKVPKSPIRELLELEPETAKFGKPEKLADGRRVRVTVEVFGVF
Dm-Dicer1 1196 VWHVYSNMMSPEIEQFSNSVPKSPIRELLELEPETAKFGKPEKLADGRRVRVTVDVFGTF
Ce-Dicer1 1198 TWQVIFHMMRGTIELCCANPPRSPIRELMEFEQSKVRFSKMERILESGKVRVTVEVVMRF
Tc-Dicer2 1189 VWRVFYKIIWREIDLFSKNVPKNVIRRLYECHTVYPPQFSK--ALEVGNQKTMVDFMGRV
Dm-Dicer2 1191 TWEVIFNLFEPELQEFTRKVPINHIRQLVEHKHAKPVFSSP--IVEGETVMVSCTCMEKV

Tc-Dicer1 1250 KGIGRNYRIAKCTAAKCALKNLKK
Dm-Dicer1 1256 RGIGRNYRIAKCTAAKCALRQLKK
Ce-Dicer1 1258 TGMGRNYRIAKATAAKRALKYLHQ
Tc-Dicer2 1247 HGFGTNKILAKRAAAKIALRALKL
Dm-Dicer2 1249 YGFGSNKDQAKLSAAKHALQQLSK
```

Dicer proteins (with Drosha)

```
Tc-Dicer1    1 HPGPSPNVLLQALTMSNANDGINLERLETIGDSFLKYAITNYLYSKYENVHEGKLSHLRS
Dm-Dicer1    1 HPGPSPSIILQALTMSNANDGINLERLETIGDSFLKYAITTYLYITYENVHEGKLSHLRS
Tc-Dicer2    1 NQSPNLCQIYQALTAAEANDIVNLERLETLGDSFLKFVASLYIIFKFPTYNEGKSTTLKG
Dm-Dicer2    1 ITPAEQGEFLAAITASSAADVFDMERLEILGDSFLKLSATLYLASKYSDWNEGTLTEVKS
Ce-Dicer1    1 GLGVSPCLLLTALTTSNAADGMSLERFETIGDSFLKFATTDYLYHTLLDQHEGKLSFARS
Tc-Drosha    1 RAFTDRSVGYTNLTLG------SNQRLEFLGDTVLQLIASEYLYKYFPEHHEGHLSLLRS
Dm-Drosha    1 RAFTDRSIGFTHLTLG------SNQRLEFLGDTVLQLICSEYLYRHFPEHHEGHLSLLRS

Tc-Dicer1   61 KQVSNLNLYRLGRRKGLGEYMIATKFTQHSIPDKSIADCVEALIGAYLIECGPRGALLFM
Dm-Dicer1   61 KQVANLNLYRLGRRKRLGEYMIATKFSQHSIPDKSIADCVEALIGAYLIECGPRGALLFM
Tc-Dicer2   61 KLVSNKNLYYLGVRKNLGGILKNSDLNKQYVGDKSIADSVEALLGAYFLSGGIQGGIKFM
Dm-Dicer2   61 KLVSNRNLLFCLIDADIPKTLNTIQFGLVTIPNKVIADTLEALLGVIVKNYGLQHAFKML
Ce-Dicer1   61 KEVSNCNLYRLGKKLGIPQLIVANKFTQQHISDKSIADAVEALIGVHLLTLGPNPTLKVM
Tc-Drosha   55 SLVNNRTQAVVCDDLGMSNYAVYNNP-KAELKTKDRADLLEAFIGALYVDQGLEFCEVFC
Dm-Drosha   55 SLVNNRTQAVVCDDLGMPKYAVYANP-KADLKTKDRADLLEAFIGALYVDKGLLYCEQFC
```

```
Tc-Dicer1  121 AWLGIRVLPQLEDGRDRSYLLQALTHASFSPNTLTDCYQRLE
Dm-Dicer1  121 AWLGVRVLPITRQLRDRSYLLQAMTHASYTPNRLTDCYQRLE
Tc-Dicer2  121 EWIGILPLSEQIQRTNRAFLLQALTHSSYSPNRITLSYERLE
Dm-Dicer2  121 EYFKICRADIDKPLKDRRYLLQALTHPSYPTNRITGSYQELE
Ce-Dicer1  121 NWMGLKVIQKDQKSKERAYLVQAFTHASYINNRVTGCYQRLE
Tc-Drosha  114 QVTLFPRLQDFIMNQDWNDPKSKLQQCCLTLRTMDGGEPDIP
Dm-Drosha  114 HVCLFPRLQLFIMNQDWNDPKSKLQQCCLTLRTMDGGEPDIP
```

dsRBM proteins

```
Dm_R2D2      1 KSAVSALQEFCARTQINLPTYSFIPGEDG----GYVCKVELLEI-----EALGNGRSKRD
Tc_R2D2      1 KTPAMVLQEFTMKRGFSPPEYILVMSKTGTHENEFHYKVNVANV-----CGLGFGRSKQV
Tc_C3PO      1 KTPISLLQELMAARGISLPVYIEEG-----FGTSFKCTVKAGGVT-----ACGYGTSKKN
Dm_Loqs      1 KTPVSILQELLSRRG-ITPGYELVQIEGAIHEPTFRFRVSFKDK-DTPFTAMGAGRSKKE
Tc_Loqs      1 KTPVSVLQELLSRRG-ATPKYELVQIEGAIHEPIFRYRVFINN----DLVATGTGRSKKD
Dm_Pasha     1 KSFVCILHEYVQHALKTQPTYEFKELQNAATPYSATVSVNNLKY------GTGYGTSKKQ
Tc_Pasha     1 KSYVCILHEYVQHALKKQPTYKFTELENAATPYAATVSINDMQY------GVGYGTSKKQ
Hs_TRBP2     1 KTPISLLQEYGTRIG-KTPVYDLLKAEGQAHQPNFTFRVTVGDT-----SCTGQGPSKKA
Hs_PACT      1 KTPIQVLHEYGMKTK-NIPVYECERSDVQIHVPTFTFRVTVGDI-----TCTGEGTSKKL
Hs_DGCR8     1 KSEVCILHEYMQRVLKVRPVYNFFECENPSEPFGASVTIDGVTY------GSGTASSKKL

Dm_R2D2     52 AKHLAASNILLRDYCVRREMPLPCIEVVQQSGTPSAPEFVACCSVASIVRYGKSDKKKDA
Tc_R2D2     56 AKHNAASKALLKDMCCEFKLPYPEFKEISDVGPPHCREFTYECCIASITTQATANTKKQA
Tc_C3PO     51 AKHESAKNALLNEYAASR--YGAKYPSYDFLTVSVDGEFFFKCSFANEESIGEGMNKKDA
Dm_Loqs     59 AKHAAARALILQEMCMQRRWPPPSYETETEVGLPHERLFTIACSILNYREMGKGKSKKIA
Tc_Loqs     56 AKHAAAKNLLLQEMCMSRRWPPPSYEMEHEEGLPHERQFTIACQVLKFKEVGTGKSKKLA
Dm_Pasha    55 AKSEAARETLILLTCLQRNYGS-DVQISQEINRTANNKNEFTMTVGKHTAKVVCKNKREG
Tc_Pasha    55 AKSEAARATLILLTCLQRNFGLNDLQISYQGNTLKNKKNQFTMTVGKHTATVVCKNKRDG
Hs_TRBP2    55 AKHKAAEVALLQELVVQKGWRLPEYTVTQESGPAHRKEFTMTCRVERFIEIGSGTSKKLA
Hs_PACT     55 AKHRAAEAAILQELAIHHGWRLPEYTLSQEGGPAHKREYTTICRLESFMETGKGASKKQA
Hs_DGCR8    55 AKNKAARATLILHECLKRNHGMGDTSIKFEVVPGKNQKSEYVMACGKHTVRGWCKNKRVG

Dm_R2D2    112 RQRAAIEMLALI
Tc_R2D2    116 KQLAAREMLEKI
Tc_C3PO    109 KQDSARKMLELV
Dm_Loqs    119 KRLAAHRMWMRL
Tc_Loqs    116 KRMAAHKMWQAL
Dm_Pasha   114 KQLASQAILQIL
Tc_Pasha   115 KQRASQAILQAL
Hs_TRBP2   115 KRNAAAKMLLRV
Hs_PACT    115 KRNAAEKFLAKF
Hs_DGCR8   115 KQLASQKILQLL
```

Argonaute proteins

```
Sp-Ago       1 DYLFFILDKNSPEPYGSIKRVCNTMLGVPSQCAISKHIL---------QSKPQYCANLGM
Tc-Ago2a     1 DLIIVVVPNSGPQ-YSLVKQAAELNVGCLTQCIKERTIA---------KLNPQIIANILL
Tc-Ago2b     1 DLIIVVVPNSGPQ-YSFVKQAAELNVGCLTQCIKERTIG---------RLNPQTVGNILL
Tc-Ago1      1 QLVVVVLPGKTPV-YAEVKRVGDTVLGMATQCVQAKNVN---------KTSPQTLSNLCL
Tc-Ago3      1 MVAVFICPTLRADRYSIIKKMCCVNIPVASQVILSKTLS-------NPQKVRTIIHKIAM
Tc-Piwi      1 TMILVILPNNSTERYSAIKKKCYVDRGIPTQMFVARNLT--------SKGVMSIATKVAI
Dm-Ago2      1 DLAIVIIPQFRIS-YDTIKQKAELQHGILTQCIKQFTVER--------KCNNQTIGNILL
Dm-Ago3      1 QMVVCICHNRRDDRYAAIKKICCSEIPIPSQVINAKTLQ-------NDLKIRSVVQKIVL
```

```
Dm-Piwi     1 KLILCLVPNDNAERYSSIKKRGYVDRAVPTQVVTLKTTK-------KPYSLMSIATKIAI
Dm-Aub      1 QIVMVVMRSPNEEKYSCIKKRTCVDRPVPSQVVTLKVIAPRQQ---KPTGLMSIATKVVI
Dm-Ago1     1 QLVVVVLPGKTPV-YAEVKRVGDTVLGMATQCVQAKNVN---------KTSPQTLSNLCL
Ce-Alg1     1 TLVVVVLPGKTPV-YAEVKRVGDTVLGIATQCVQAKNAI---------RTTPQTLSNLCL
Ce-Alg2     1 QLIVVVLPGKTPI-YAEVKRVGDTVLGIATQCVQAKNAI---------RTTPQTLSNLCL
Ce-Prg1     1 HMLVVMLADDNKTRYDSLKKFLCVECPIPNQCVNLRTLAGKSKDGGENKNLGSIVLKIVL
Ce-Prg2     1 HMLVVMLADDNKTRYDSLKKYLCVECPIPNQCVNLRTLAGKSKDGGENKNLGSIVLKIVL
Ce-RDE1     1 VLMFIIISKRQLNAYGFVKHYCDHTIGVANQHITSETVTKALASLRHEKGSKRIFYQIAL
Ce-Ergo     1 RATVYSGNNNEYNDYNVLKYLADNKYGIHTQGILEKSLGVVGPS-PKNCALTRLMVEKVL
Ce-PPW1     1 TQLLFFVVKSRYNYHQQIKALEQKYDVLTQEIRAETAEK--------VFRQPQTRLNIIN
Ce-PPW2     1 TFVLCITEDNITCLHQKYKFIEHHTQMIVQDMKLSKALSVVN------ASKKLTLENVIN

Sp-Ago     52 KINVKVGGINCSLIP-KSNPLGNVP-TLILGGDVYHPGVGATG-VSIASIVASVD-LNGC
Tc-Ago2a   51 KINSKLNGTNHILS--SRLPIMSRP-CIIMGADVTHPGPDAKDVPSVAAVTASHD-PNAF
Tc-Ago2b   51 KINSKMNGTNHRLSPNSRPLIMKRP-CMIMGADVTHPSPDARDIPSVAAVTASHD-PNAF
Tc-Ago1    51 KINVKLGGINSILVPSIRPKIFNEP-VIFLGADVTHPPAGDNKKPSIAAVVGSMD-AHPS
Tc-Ago3    54 QITCKLGGTLWSVKIPVSG-------WMVCGIDVYHGA--NNQ--SVCGFVASIN-GSMT
Tc-Piwi    53 QMNCKIGGAPWCVPIPLSG-------LMVVGYDVCRDT--VNKKKSFAGIVGSLD-KNIS
Dm-Ago2    52 KINSKLNGINHKIKDDPRLPMMKN--TMYIGADVTHPSPDQREIPSVVGVAASHD-PYGA
Dm-Ago3    54 QMNCKLGGSLWTVKIPFKN-------VMICGIDSYHDP--SNRGNSVAAFVASIN-SSYS
Dm-Piwi    54 QLNCKLGYTPWMIELPLSG-------LMTIGFDIAKST--RDRKRAYGALIASMDLQQNS
Dm-Aub     58 QMNAKLMGAPWQVVIPLHG-------LMTVGFDVCHSP--KNKNKSYGAFVATMDQKESF
Dm-Ago1    51 KINVKLGGINSILVPSIRPKVFNEP-VIFLGADVTHPPAGDNKKPSIAAVVGSMD-AHPS
Ce-Alg1    51 KMNVKLGGVNSILLPNVRPRIFNEP-VIFFGCDITHPPAGDSRKPSIAAVVGSMD-AHPS
Ce-Alg2    51 KMNVKLGGVNSILLPNVRPRIFNEP-VIFLGCDITHPAAGDTRKPSIAAVVGSMD-AHPS
Ce-Prg1    61 QMICKTGGALWKVNIPLKN-------TMIVGYDLYHDS--TLKGKTVGACVSTTS-NDFT
Ce-Prg2    61 QMICKTGGALWKVNIPLKS-------TMIVGYDLYHDS--TLKGKTVGACVSTTS-NDFT
Ce-RDE1    61 KINAKLGGINQEISPEEKERRKTMPLTMYVGIDVTHPTSYSGIDYSIAAVVASINPGGTI
Ce-Ergo    60 GKVGTGAHKTWTIFTDPAKP------TLVLGIDVSHPSTRDRETMSAATVVGNID-LDVT
Ce-PPW1    53 KTNMKLGGLNYAIGSEAFNKPN----RLIVGFVTSQRVGGNPDYPISVGFAANMLKHHQK
Ce-PPW2    55 KTNVKLGGSNYVYLDTKNFLQ----EHLIIGVGISSPPPGTKYILNPTIVGFAYN-GNGK

Sp-Ago    108 KYTAVSRSQPRHQEVIEGMKDIVVYLLQGFRAMTK-QQPQRIIYFRDGTSEGQFLSVIND
Tc-Ago2a  107 QYNICWRLQPPKVEIIEDLCAITVEQLMFFYRKTR-HKPETIVFFRDGVSEGQFAEVRRA
Tc-Ago2b  109 QYNICWRLQPPKVEIIEDLCNITVEQLKFFYQKTG-FKPESIVFFRDGVSEGQFKQVQRA
Tc-Ago1   109 RYAATVRVQQHRQEIIQELSSMVRELLIMFYKSTGGYKPHRIILYRDGVSEGQFLQLLQH
Tc-Ago3   102 KYFSKAMFQD--GEIGDYFKMPFRQMLQAAKDRE-GAFPSKVIVFRDGVGDGQLEHCRKY
Tc-Piwi   103 RFYNICCEHKMEEELSDNFAAAVVLLCKQYKEQN-GHYPERILIYRDGVGEGQLPFVVEH
Dm-Ago2   109 SYNMQYRLQRGALEEIEDMFSITLEHLRVYKEYRN-AYPDHIIYYRDGVSDGQFPKIKNE
Dm-Ago3   104 QWYSKAVVQTKREEIVNGLSASFEIALKMYRKRN-GKLPTNIIIYRDGIGDGQLYTCLNY
Dm-Piwi   105 TYFSTVTECSAFDVLANTLWPMIAKALRQYQHEH-RKLPSRIVFYRDGVSSGSLKQLFEF
Dm-Aub    109 RYFSTVNEHIKGQELSEQMSVNMACALRSYQEQH-RSLPERILFFRDGVGDGQLYQVVNS
Dm-Ago1   109 RYAATVRVQQHRQEIIQELSSMVRELLIMFYKSTGGYKPHRIILYRDGVSEGQFPHVLQH
Ce-Alg1   109 RYAATVRVQQHRQEIISDLTYMVRELLVQFYRNTR-FKPARIVVYRDGVSEGQFFNVLQY
Ce-Alg2   109 RYAATVRVQQHRQEIITDLTYMVRELLVQFYRNTR-FKPARIVVYRDGVSEGQLFNVLQY
Ce-Prg1   111 QFYSQTRPHENPTQLGNNLTHFVRKALKQYYDSNDQTLPSRLILYRDGAGDGQIPYIKNT
Ce-Prg2   111 QFYSQTRPHENPTQLGNNLTHFVRKSLKQYYDNNDKTLPSRLILYRDGAGDGQIPYIKNT
Ce-RDE1   121 YRNMIVTQEECRRERTDILEAKFVKLLREFAENNDNRAPAHIVVYRDGVSDSEMLRVSHD
Ce-Ergo   113 EFRASSRIQDTGVECLIDFSKEIDERIGEFIDHT-GKRPAHIVVYRDGLSEGDFQKYLFE
Ce-PPW1   109 FAGGYVYVHRDRDVFGSIIKDTILAIFKTCTEQR--GRPDDILLYFNGVSEGQFSMINEE
Ce-PPW2   110 QEFSGD-VLNAAQETIAPIEDIVSYSIKGYKKFHDGKAPKRITIYRSGSSEGNHGPIISY

Sp-Ago    167 ELSQIKEACHSLSP------KYNPKILVCTTQKRHHARFFIKNKSDG--DRNGNPLPGTI
Tc-Ago2a  166 EISAIHQACKKLQRE-----GYEPRITFLVVQKRHHTRLFPTNPRDSE-DRNNNVPAGTC
Tc-Ago2b  168 EIAAIQKACKMLQKD-----DYEPKITFLVVQKRHHTRLFPTNPRDSE-DKNNNVPAGTC
Tc-Ago1   169 ELTAIREACIKLES------DYKPGITFIVVQKRHHTRLFCADKKEQS-GKSGNIPAGTT
Tc-Ago3   159 EITQLQEVIKELNIE--------TTITFVVVQKRINTRIFRTVNETN----FENPPSGTV
Tc-Piwi   162 EVANIKRKLQEEIYINGEVK-----MAFVVVSKRINTRIFTEKD---------NPPPGTV
```

```
Dm-Ago2    168 ELRCIKQACDKVG--------CKPKICCVIVVKRHHTRFFPSGDVTTS-NKFNNVDPGTV
Dm-Ago3    163 EIPQFEMVCGNR-----------IKISYIVVQKRINTRIFSGS-GIH----LENPLPGTV
Dm-Piwi    164 EVKDIIEKLKTEYARVQLSP---PQLAYIVVTRSMNTRFFLNGQ---------NPPPGTI
Dm-Aub     168 EVNTLKDRLDEIYKSAGKQEG--CRMTFIIVSKRINSRYFTGHR---------NPVPGTV
Dm-Ago1    169 ELTAIREACIKLEP------EYRPGITFIVVQKRHHTRLFCAEKKEQS-GKSGNIPAGTT
Ce-Alg1    168 ELRAIREACMMLER------GYQPGITFIAVQKRHHTRLFAVDKKDQV-GKAYNIPPGTT
Ce-Alg2    168 ELRAIREACVMLES------GYQPGITFIAVQKRHHTRLFAADKADQV-GKAFNIPPGTT
Ce-Prg1    171 EVKLVRDACDAVTDKAAELSQEKIKLAFIIVTKRVNMRILKQGSLDN----AINPQPGTV
Ce-Prg2    171 EVKLVRDACDAVTDKAAELSQEKIKLAFIIVTKRVNMRILKQGSSKS----AINPQPGTV
Ce-RDE1    181 ELRSLKSEVKQFMSERDGEDPE-PKYTFIVIQKRHNTRLLRRMEKESKETGIVNPSSGTT
Ce-Ergo    172 ERVCIEERCLKIDTSFQP------SITYIVVTKRHHTQFFLEDPSQGYESQGYNVLPGTL
Ce-PPW1    167 FSARVKEACMAFQKEGTP--PFRPHITIIASSKAHNERLYKSDKGR-----IVNLEPGTV
Ce-PPW2    169 EVPLARVAMRNFSP--------DTQLLYIVVSKEHTYRFFKKESSAPPKPWELNIGPGLT

Sp-Ago     219 IEKHVTHPYQYDFYLISHPSLQGVSVPVHYTVLHDEIQMPPDQFQTLCYNLCYVYARATS
Tc-Ago2a   220 VDTHITNPMMQDFYLVSHASIQGVAKPTKYCTLWDDNNMSNDDIEELTYYLCHMFTRCNR
Tc-Ago2b   222 VDTHITNPRMQDFYLVSHASIQGVAKPTKYCTLWDDNNMNNDDIEELTYHLCHMFTRCNR
Tc-Ago1    222 VDVGITHPTEFDFYLCSHQGIQGTSRPSHYHVLWDDSHLDSDELQCLTYQLCHTYVRCTR
Tc-Ago3    207 VDNMVTRRQFYDFFLVPQSVRQGTVNPTHYVVLVDEGNIKPDHLQRLAYKLCHLYYNWSG
Tc-Piwi    208 VDDVITLPERYDFYIVSQCVRQGTVAPTSYNVIEDSMGLPPEKLQYLTYKLTHMYYNWSG
Dm-Ago2    219 VDRTIVHPNEMQFFMVSHQAIQGTAKPTRYNVIENTGNLDIDLLQQLTYNLCHMFPRCNR
Dm-Ago3    207 VDQHITKSNMYDFFLVSQLVRQGTVTPTHYVVLRDDCNYGPDIIQKLSYKLCFLYYNWAG
Dm-Piwi    212 VDDVITLPERYDFYLVSQQVRQGTVSPTSYNVLYSSMGLSPEKMQKLTYKMCHLYYNWSG
Dm-Aub     217 VDDVITLPERYDFFLVSQAVRIGTVSPTSYNVISDNMGLNADKLQMLSYKMTHMYYNYSG
Dm-Ago1    222 VDVGITHPTEFDFYLCSHQGIQGTSRPSHYHVLWDDNHFDSDELQCLTYQLCHTYVRCTR
Ce-Alg1    221 VDVGITHPTEFDFYLCSHAGIQGTSRPSHYHVLWDDNNLTADELQQLTYQMCHTYVRCTR
Ce-Alg2    221 VDVGITHPTEFDFFLCSHAGIQGTSRPSHYHVLWDDNDLTADELQQLTYQMCHTYVRCTR
Ce-Prg1    227 VDTTVTRPERMDFYLVPQFVNQGTVTPVSYNIIHDDTDLGPDKHQQLAFKLCHLYYNWQG
Ce-Prg2    227 VDTTVTRPERMDFYLVPQFVNQGTVTPVSYNIIHDDTGLGPDKHQQLAFKLCHLYYNWQG
Ce-RDE1    240 VDKLIVSKYKFDFFLASHHGVLGTSRPGHYTVMYDDKGMSQDEVYKMTYGLAFLSARCRK
Ce-Ergo    226 IEDAVTTNKYYDFFLSTQIGNEGCFRPTHYYVLHDTWTGKPDFWPTVTHALTYNFCRSTT
Ce-PPW1    220 VDHTIVSNVYTEWYHASAVARQGTATKFTLIFTTKAGQAEPLHLEQLTNDLCYDHQIVFH
Ce-PPW2    221 VDYGVTNPACKQFFLNSHMTLQGSAKTPLYTVLADDRNIGMSALEEFTFNLCHLHQIVGL

Sp-Ago     279 AVSLVPPVYYAHLVSNLARYQDV
Tc-Ago2a   280 SVSYPAPTYYAHLAAARAKVYVE
Tc-Ago2b   282 SVSYPAPTYYAHLAAARAKVYIE
Tc-Ago1    282 SVSIPAPAYYAHLVAFRARYHLV
Tc-Ago3    267 TIRVPAPCLYAHKLAAIVGQYIK
Tc-Piwi    268 TVRVPAPCQYAHKLAFMVSQYIH
Dm-Ago2    279 SVSYPAPAYLAHLVAARGRVYLT
Dm-Ago3    267 TVRIPACCMYAHKLAYLIGQSIQ
Dm-Piwi    272 TTRVPAVCQYAKKLATLVGTNLH
Dm-Aub     277 TIRVPAVCHYAHKLAFLVAESIN
Dm-Ago1    282 SVSIPAPAYYAHLVAFRARYHLV
Ce-Alg1    281 SVSIPAPAYYAHLVAFRARYHLV
Ce-Alg2    281 SVSIPAPAYYAHLVAFRARYHLV
Ce-Prg1    287 TVRVPAPCQYAHKLAFLTAQSLH
Ce-Prg2    287 TVRVPAPCQYAHKLAFLTAQSLH
Ce-RDE1    300 PISLPVPVHYAHLSCEKAKELYR
Ce-Ergo    286 TVALPAPVLYAHLAAKRAKETLD
Ce-PPW1    280 PVGLPVPLYIADRYSQRGAMVLA
Ce-PPW2    281 PTSIPTPLYVANEYAKRGRNLWN
```

RdRP families

```
Branchiostoma     1 WRCALSRGYKVSDRTASALHQLADTELKHYVYARKVVVTPTQMLFLKPEPIVENRVVREY
Schizosachara     1 PDYKHRCSLINDKENLKKLSKERAKKFLSSIHIKKLYVTPTTLRIVEDSLEAGNRVIRNF
```

```
Neurospora       1 FDVRYQLEVCISQGRARLILEYVADEYAHCAWVRRVTITPTRIYFSTPCVEPTNRVIRQW
Aspergillus      1 FSVRYQLEVCISNGQATKLLEHVATQKRYCCYIRSARVTPSTVYYETPSMDMSNRIIRHY
Coccidioides     1 FTLKFQIAKLVWNGNLEYPSPDTHESDVNNVKVHRAQVTPSGTYLYGPNIETKNRVLRQY
Arabidopsis      1 FEILFKLNTLVQNACLEKLFHLGECCYEGLVYMYRVQVTPARVYFSGPEVNVSNRVLRHY
Nicotiana        1 YKILFKISSLIQHGALEKLYYLKECCYDGLVYVRRVVVTPCKVYFCGPEVNVSNRVLRNY
Hordeum          1 YEILFKINHLVQNGALEKMSYLKKTCLNGLVYVYRVQITPAKVYFYGPEINVSNRVVRNY
Solanum          1 YKILFKISSLVQHGALEKLYYLKECCYDGLVYVRRVLVTPCKVYFCGPEVNVSNRVLRNY
Ce-ego1          1 FAITYLLECLISRGGLEDLVHMIDGRKRGYQRVRKIIFTPTRVIYVAPETLMGNRVLRRY
Ce-RRF1          1 FSITYLIECLLSRGALEDLVHLIDGRKRGYQRVRKVIFTPTRVIYIAPETIMGNRVLRKF
Ce-RRF3          1 FRANYALQALITRGTLEQLLNAFDERRQNCVSVAKVIVTPSRILLMAPEVMMVNRVVRRF
Dyctiostelium    1 YESSLELNCFIQGGIITSLISKRTKFINEFTMIRDVFITPSRTIFIQPTLQRSCRAVRKF
Tetrahymena      1 FNLSYSILTLISHNGIQYYYNQKKEINRKVIATRRVSITPSGLIYNLKELELSNRVLRIF

Branchiostoma   61 G--VDNFIRVAFRDEDFSKLAVTSRVKDVLERGIRIGERHFKYLGSSNSQMREHGCWMYA
Schizosachara   61 KDFANRFMRVQITDEYYKQKILYSRIQQLLTYGIKVGNQIYEFLAFGNSQLREHGAYFFA
Neurospora      61 KHAQDYFIRIQFTDEVLEGRILFLRVYRVLEKGVAMGPWHWKFLAFGNSQIREAGAFMFC
Aspergillus     61 IEYADRFLRVRFTDEKLLGRIVFTRVKRALANGIVIGDRRYEFLAFGNSQFREHGAYFFA
Coccidioides    61 NDHINNFLRVEFVDETGDPVYFHERFKGVLKQGIKIGGCHFSFLGFSHSSLRSQTCWFVA
Arabidopsis     61 SKYINNFLRVSFVDEDLEKVRLYDRIYSVLRDGIVIGDKKFEFLAFSSSQLRENSAWMFA
Nicotiana       61 SEDINNFLRVSFVDEEWEKIHIYERILSTLRNGFIIGDKRFEFLAFSSSQLRDNSVWMFA
Hordeum         61 AADLDNFLRISFVDEDCEKLRLYNRVLSVLSNGITIGDKHFDFLAFSSSQLRDNSAWMFA
Solanum         61 SEDIDNFLRVSFVDEEWEKLYIYERILSTLRKGFVIGDKKIEFLAFSSSQLRDNSVWMFA
Ce-ego1         61 DHDGTRVLRITFRDDDNQKMRLEKTVNQYLKNGITVAGRNFGYLGSSNSQMRDNGAYFME
Ce-RRF1         61 DKDGTRVLRVTFRDDNNKKMRLDRTANKYLEHGVRIANREYGFLGCSNSQMRDNGAYFMM
Ce-RRF3         61 G--PDYALRCVFRDDNLGRLAVTEGIYLTLKNGIQVADRVYSFLGWSNSQMRDQGCYLYA
Dyctiostelium   61 G--SSNFIMVKIVNESLEPLQHENRIKPILEGGILVGGKIYSYAGNSNSQLREYSSWFVS
Tetrahymena     61 QDSTDSFIRGTFQDDSLENAK---RYFRQKLKSFKVLNWEFINFGWSASQLRNSSSWFFH

Branchiostoma  119 AVDTTSTSDIRGWMGDLSHERCVATYVSRLGQFFSSSRNAT-HTFSDGIGKISVPLAKKV
Schizosachara  121 SGSDLNAKQIREWMGDFSEINSVSKYAARMGQCFSTTKEINNHCFTDGVGMASLSVIRRL
Neurospora     121 EQSNLTGDMMRAWMGRFSHIKVIAKYAARLGQCFSTTRLVDGFCFTDGVGKISPLLAKIV
Aspergillus    121 PLPNLTAANIRAWMGHFDDIRNVAKHAARLGQCFSTTRAINGYRFSDGVGRISKFLAQMV
Coccidioides   121 PFTRFDAQSIIQGLGSFSHIYSPAKLAARIGQTFSETQTSNGRVFSDGVGTFSPRLLYKI
Arabidopsis    121 PIDRITAAHIRAWMGDFDHIRNVAKYAARLGQSFSSSRETTRYVFSDGIGKISAEFARKV
Nicotiana      121 SRPGLTANDIRTWMGDFRQIRNVAKYAARLGQSFGSSRETTNYIFSDGIGKISADFAHRV
Hordeum        121 SRPGLSASDIREWMGNFRNIRNVAKYAARLGQSFSSSTETTEYVFSDGIGTISADFADEV
Solanum        121 SRPGLTANDIRTWMGDFSQIKNVAKYAARLGQSFGSSKETTSYVFSDGIGKISADFARRV
Ce-ego1        121 KYSNPKIQAARKNLGRFETIDNIPKMMARLGQCFTQSRLSDEYTFSDGVGMMSYRFAQMV
Ce-RRF1        121 RFTKPKIDEVRFQLGRFSEIENVPKLMARLGQCFTQSRLTSEYTFSDGVGMMSYQFAQEV
Ce-RRF3        119 PRVTGTVEDIRVWMGDFRDAISVPKMMSRMGQCFTQAQPTNKYCFSDGCGRISIKLATHI
Dyctiostelium  119 NQI---THTVKIWSG-IEHVDNVRKFFRCIGLMFSTTIPTNTHVFTEGCGEIGPELAKHL
Tetrahymena    118 ETEVLKKQDVITRIGDFSGINPPAKKSARIGQCFSSSTPYKKKVFTDGIGTISQDLIRKI

Branchiostoma  178 PSAFQIRYAGCKGVLAQDPTLRIQIRGSMKKFETLEVTSGRIMMGTMDETGKLQYGQVFI
Schizosachara  181 PSAFQFRMGGYKGVLSLAPPTLVFPRRSQDKFKKVSVPKGAYLLGVADETGTLKGHYDDA
Neurospora     181 PSAYQFRMGGCKGVLVTWPDVEVHIRKSQEKFVRLNVEKSAFVLGCVDETGTLKGHMKVI
Aspergillus    181 PSVFQFRLGGCKGILTVSPQAEVHIRKSQAKFTKITIDKGACVFGCMDETGTLKGYFHEK
Coccidioides   181 PTVFQIRFAGAKGMVSLDTRLQLRLRESMVKFSRIRVPEAVTLYGIMDETGYLNEGEIFC
Arabidopsis    181 PSAFQIRYGGYKGVVAVDPNSKLSLRKSMSKFERIFISGGRSMMGCLDETRTLEYGQVVV
Nicotiana      181 PSSFQIRYGGYKGVVAVDPYSKLSLRKSMLKYERIFIPNGRTMMGCLDESRTLEYGQVFV
Hordeum        181 PSAFQIRYGGYKGVVAIDPTSKLSLRKSMSKFQRIFVPKGRAMMGCLDETRTLKYGQVFI
Solanum        181 PSAFQIRYGGYKGVVAVDPYSKLSLRKSMSKYERIFIPNGRAMMGCLDESRTLEYGQVFV
Ce-ego1        181 PSCFQFRFRGMKGVISIEPLLNCMFRPSQIKFIQIPIPCGRSMLGVVDETGRLQYGQIFV
Ce-RRF1        181 PSCFQIRFRGNKGVIAIEPFLKCLFRPSQIKFQQIQIPSGRSMLGVVDETGRLQYGQIFV
Ce-RRF3        179 PACFQVRFKGFKGILVIDPTIKVIFRKSQQKFGKIFLPPGRSMYGVVDETGLLQYGQVFI
Dyctiostelium  175 TCAYQVRIGGNKGMLVVNNQAGIYIRPSMVKFNHIEIKDSRMLLGVCDPTNSLPPNTVFV
Tetrahymena    178 MCAIQVRYGGAKGVLTIDYKSTIQLRESMIKYKNIFLKKSARLMGVIDEYNVLEQDEVFA
```

```
Branchiostoma   238 QYSVVHQGQVTVTKNPYFHPGDMRKFTAVDVPELRHMVDCIVFPSRGPRPHPDEMSGSDL
Schizosachara   241 VLSRVIVGLCIVARNPSLHPGDVRVCKAVRCDELMHLKNVIVFPTTGDRSIPAMCSGGDL
Neurospora      241 EDWRVITGTCVVGRNPSLHPGDIRVVEAVDVPALRHLRDVVVFPLTGDRDVPSMCSGGDL
Aspergillus     241 IPDEVIQGMCILARNPSLHSGDIRVVKAVDVPALRHLQDVVVLPQTGDRDIASMCSGGDL
Coccidioides    241 TFLILVRDQVIVTRAPALHPGDVQYAKAVDVPPLRSLHNCVIFSQHGFRDLPSMLSGGDL
Arabidopsis     241 QYSFIITGPVVVAKNPCLHPGDVRVLQAVNVPALNHMVDCVVFPQKGLRPHPNECSGSDL
Nicotiana       241 QFSFILKGNVVVAKNPCLHPGDIRVLRAVDVPALHHMVDCVVFPQKGKRPHPNECSGSDL
Hordeum         241 QASSVVTGKVIVAKNPCIHPGDIRILQAVHSPLLGHMVNCVVFPQLGPRPHPNECSGSDL
Solanum         241 QFTFILKGNVVVAKNPCLHPGDIRVLRAVDVPALHHMVDCVVFPQKGKRPHPNECSGSDL
Ce-ego1         241 QYTQVLTGTVLLTKNPCIVAGDVRIFEAVDIPELHHMCDVVVFPQHGPRPHPDEMAGSDL
Ce-RRF1         241 QYTEIVTGTVLLTKNPCIVPGDVRIFEAVDIPELHHMCDVVVFPQHGPRPHPDEMAGSDL
Ce-RRF3         239 QYSILKTGKVLITKNPCHVPGDVRVFDAVWQPALAHLVDVVVFPQHGPRPHPDEMAGSDL
Dyctiostelium   235 QLEKVIEGLVMVIKNPCTHPGDVRYLKAVDNL-LRHLRNVLVFSTKGDVPNFKEISGSDL
Tetrahymena     238 YISFQVEGDVIVVKNPCLHPGDIRKLKAVTLEPFKKLRNILIFPQKGKEPITSQIAGSDL

Branchiostoma   298 DGDMYFVTWREGLILPRENRPAMDFTAQKRVLHRPVQESDMIEFFAEYIGSNQLGLIANA
Schizosachara   301 DGDEYTVIWDQRLLPKIVNYPLESSPKKIDFLEGKPLIDSVKEFFVNYIKYDSLGLISNA
Neurospora      301 DGDDFFVIWDPLLIPKERSHPPMISEPIGKELATEPTVNNLITFFVLYMKYNNLPLIAHA
Aspergillus     301 DGDDYVVIWDQDLIPKDWFRPPMNYTTNARDLDRDVTVDEITSFFVTYMKNDCLPTIAHA
Coccidioides    301 DGDLYNVIYDERLMPPCTYPP-ANYPKVEKLLDREVVRDDIIDFFVTFMQQDQLGRIATI
Arabidopsis     301 DGDIYFVCWDQELVPPRTSEPMDYTPEPTQILDHDVTIEEVEEYFANYIVNDSLGIIANA
Nicotiana       301 DGDIYFVCWDPDLIPPRQVQSMDYTPAPTTQLDHDVTIEEVEEYFTNYIINDSLGIIANA
Hordeum         301 DGDIYFVSWDPDLIPTRMVAPMDYTPAPTETLDHDVMIEEVHEYFTNYIVNESLGIIANA
Solanum         301 DGDIYFVCWDQDMIPPRQVQPMEYPPAPSIQLDHDVTIEEVEEYFTNYIVNDSLGIISNA
Ce-ego1         301 DGDEYSIIWDQQLLLDKNEDPYDFTSEKKASFKEDEIDDLMREFYVKYLKLDSVGQISNS
Ce-RRF1         301 DGDEYSVIWDQELLLERNEEPFDFAVEKKVPYDREKLDVLMREFYVTYLKLDSVGQISNS
Ce-RRF3         299 DGDEYSIIWDQEMLLDYNEEA-MVFPSSAAEEDKEPTTDDMVEFFLRYLQQDSIGRMSHA
Dyctiostelium   294 DGDRYFFCYDKSLIGNRSKSEYLGDETVNNDKKANNDPFALSSMYSTNVERQELGKMYNS
Tetrahymena     298 DGDCYFICWDQRFIPLEQKEPYDEDDNSKKQISQEDDNEQMLDFFIDFINNENLGKIDNS

Branchiostoma   358 HLVHADKEDQGIFSQKCIDLAQLHSDAVDAPKTGELKGDLRPEVYPDFMMKSDKPQYSER
Schizosachara   361 WKAWADNNPEGIFGNVCLELAEMHSKAVDFAKSGKMQAKYHPKRYPDFMQKTKTRSFSET
Neurospora      361 HLATADAEVEGVKSPKCLELASLHSMAVDYVKTGEFPRRLDPKTWPHFMEKNRHTYHSVT
Aspergillus     361 HLAWADRLDNGVDEEKCIRLAQLHSDAVDYNKNGHMTRNLEPKLWPHFMEKKEKVYKSGK
Coccidioides    360 HQAIADQRPAGTLDQDCLLLAELHSVAVDFSKSGLTRIPKRPRYYPDFMAPSRIKIASIE
Arabidopsis     361 HTAFADKEPLKAFSDPCIELAKKFSTAVDFPKTGVIPQHLYVKEYPDFMEKPDKPTYSKN
Nicotiana       361 HVVFADREPDMAMSDPCKQLAQLFSIAVDFPKTGEIPSQLRPKEYPDFMEKPDKPTYSEK
Hordeum         361 HVVFADRQSLKAESTQCIKLAELFSIAVDYPKTGQIPHELHVKEYPDFMEKLDRATYSEG
Solanum         361 HVVFADREPDMAMSDPCKKLAELCSIAVDFPKTGEIPSQLRPKEYPDFMDKPDKTTYSER
Ce-ego1         361 HLHNSDQYGLNAR--VCMDLAKKNCQAVDFTKSGEMIPPERAERVPDYHMGNHTPMYSPR
Ce-RRF1         361 HLHNSDQYGLNSR--VCMDLAKKNCQAVDFTKSGEVIPPENPERIPDFHMGNRSPMYSPR
Ce-RRF3         358 HLAYA--DLHGLFHENCHAIALKCAVAVDFPKSGPLSSFEQCEMTPDYMMSGGKPMYSTR
Dyctiostelium   354 HLAIS--DLFGANHEFSIQISKECFKEIDYPKTGEANVWLKTKGYPHYMQRETRVYYSKT
Tetrahymena     358 HLVFADKSPLYAEDPKCLKLASLHAQAVDYAKTGQLPKNLAIKEYPDFMEKDDKPSYSQS

Branchiostoma   418 IIGRLFRKCQELVREGFDSYIEDAQTQLNIYNNMMMSIMSLYGITSEGEVVSGCILKVKQ
Schizosachara   421 AVGKIFRYAARKLPRFKTEYLNVAEEVKKHYDNDLRSIMARFDISTEYEVYTAFILFKDD
Neurospora      421 ALGKLYDMVKRKCRALRDGTLAKARRIKSQYDTAMRRVMCQLEIATEFEVWTAFVMSKPR
Aspergillus     421 ILGQLYDAVERIP--VAEDLYQFARDMKKDYDIALRRIMAQHEIKTEFEVWSTFVLSHAN
Coccidioides    420 VVEEEQYLAEDINEQEFLNAVRNASKLRRDDNTLLNAIWDYVASETEGFQWDHLVEDGRK
Arabidopsis     421 VIGKLFREVKEMEVDGFEEYVDEAFYQKANYDFKLGNLMDYYGIKTEAEILSGGIMRMSK
Nicotiana       421 VIGKLFQKVKNMIVDGFEDYIDEAFYYKSEYDNKLGNLMDYYGIKTEAEILSGGIMKASK
Hordeum         421 VIGKLYREIKKLIVDGYQDYITEAVWFKEEYDFKLGNLMEHYGINSEAEIISGCILKMAK
Solanum         421 VIGKLFRKVKDLEVDGFEDYIDEAFDYKTEYDSKLGNLMDYYGIKTEAEILSGGIMKTSK
Ce-ego1         419 LCGKLFREFKAIKIDGYTEYMASAKNDLARYNAQLRSMMENYGIKTEGEVFSGCIVDMRN
Ce-RRF1         419 LCGKLFREFQAIFVTGFERYMESAQKQLSSYNGQLRSIMENYGIRSEGEIMSGCIVEMRN
Ce-RRF3         416 LNGQLHRKARKDVFFGNEIKLVQTLTLRDEYVDRMQQLLDEYGIEDEASVVSGHAASIKR
```

```
Dyctiostelium   412 IMGKMYDQIDQNLVDDYEIYLNSAKILYSQYKLQVHSLLRHYSAESEESIMIGFLDQGFI
Tetrahymena     418 ILGILYRQVKDENKTYLEQEIDFAIEANNQWNIEINNLKQFFGAQNEFEIYTGNFSKLIK

Branchiostoma   478 RLKNERFEVAEYVTLWRKTRAEFFKQFDVLKKASAWYMVTYSDSPKLLSFPWVVYDVLAS
Schizosachara   481 LVNEYGLREEVSFLLKKKYTQEYLEKCALNSAVAATYDVTYDQTEVLISFPYLFSSRLCQ
Neurospora      481 VYKLQDNVGRESSALKQHFKDQCKKEAGGLSFVSAMYRVTYEETMPLVSFPWLFWDKLGE
Aspergillus     479 MYKFHEEIGMLSSSLREGFKRECYDKVGGAPLALAMYRITHEEQLPLISFPWIFPRVLGQ
Coccidioides    480 VYEDKLREIMQQYTPWKSSLTEVEVVMGSNSKQSRRDKEASQSTPLSAQLPLFGTHYMRS
Arabidopsis     481 SKRRDAESIGRAVRALRKETLSLFNASEESAKASAWYHVTYHSRDHFLSFAWCVYDKLVR
Nicotiana       481 TRRKDAEAIGVAVRCLRKEARAWFKRRSDLAKASAWYHVTYHHRDHFISFPWCVYDQLIQ
Hordeum         481 NKKSDADAIRLAVKSLRKEARSWFSEMGSVAKASAWYHVTYHPRPHLISFPWCVYDKLIL
Solanum         481 TRRKDAEAISIAVRALRKEARTWFKRHNDLPKASAWYHVTYHPRDHFISFPWCVYDQLIQ
Ce-ego1         479 RKDQDDMSFFNTNQMIETKLTNLFKKYREEGNTEAFSRYGRDSENKKLSFAWLAYDVIAK
Ce-RRF1         479 RKDQDDMSFYNTNQMIETKMTSLVCKFRETVKCTLLPNAYDNGEVRKLSFAWIAYDVIAK
Ce-RRF3         476 LERDDYSFYHTDKKLYAVFRAKFFEEFGGKNTRLKCTKAMHEKRCIGQSLPWVAWDALCD
Dyctiostelium   472 SSKDIKGEMKNDYKIQQTFENEFLKEFGEEKKVSAWYHVAYSDNKKKDTLFPLYESRLMI
Tetrahymena     478 VKKMSAEIVQQRIAIKNRFELLFFKNLDEKIRATLWYIICYLRKDSLLGQTFTFLSQQKY

Branchiostoma   538 LKKI
Schizosachara   541 LSRK
Neurospora      541 LARA
Aspergillus     539 IATG
Coccidioides    540 IVDR
Arabidopsis     541 IKKT
Nicotiana       541 IKKA
Hordeum         541 IKQK
Solanum         541 IKKE
Ce-ego1         539 VKQD
Ce-RRF1         539 VKET
Ce-RRF3         536 LRRQ
Dyctiostelium   532 VKEL
Tetrahymena     538 FGET
```

Eri-1-like nucleases

```
Ce_eri1           1 DYLIAIDFECTCVEIIY---DYPHEIIELPAVLIDVREMKIIS--EFRTYVRPVRNPKLS
Ce_M02B7.2        1 DYLLVLDFEATCQDNWKGPMHPVQEIIEFPVVQLSTADWSEIR--RFHQYIKPTEFPRLT
Hs_3hExo          1 DYICIIDFEATCEEGNPP--EFVHEIIEFPVVLLNTHTLEIED--TFQQYVRPEINTQLS
Hs_NP542394       1 DYLIVIDFESTCWNDGKHH-HS-QEIIEFPAVLLNTSTGQIDS--EFQAYVQPQEHPILS
Hs_Pint1          1 HYFLVLDFEATC-DKPQ---IHPQEIIEFPILKLNGRTMEIES--TFHMYVQPVVHPQLT
Tc_Snp            1 DYLLVLDFEATCWSNGDPR-KNPAEIIEFPVVLYDVKNAKIIA--EFQQYVMPVENPKLS
Dm_Snp            1 SYVIAVDFEATCWEKQAPPEWREAEIIEFPAVLVNLKTGKIEA--EFHQYILPFESPRLS
Urchin_1          1 DYLCVIDVEATCQEINPV--DYIHEIIEFPIVLLNTKTLQIVN--TFDAFCKPVINPQLS
Urchin_2          1 SYLIVIDFESTCWKDKKN---TSQEIIEFPAVLLNTTTGQLES--EFQQYVMPDEHPVLS
Urchin_3          1 DYFLVLDFEATCLDNER---IEPQEVIEFPVLKVSGKTFETEA--TFHQYVEPQVH-GVG

Ce_eri1          56 EFCMQFTKIAQETVDAAPYFREALQRLYTWMRKFN--------------------LGQKN
Ce_M02B7.2       59 SFCTSLTRIIQEMVDEKPKLPEVLSEFDSWLKEDS--------------------RLK-Q
Hs_3hExo         57 DFCISLTGITQDQVDRADTFPQVLKKVIDWMKLKE--------------------LGTKY
Hs_NP542394      57 EFCMELTGIKQAQVDEGVPLKICLSQFCKWIHKIQQQKNIIFATGISEP------SASEV
Hs_Pint1         55 PFCTELTGIIQAMVDGQPSLQQVLERVDEWMAKEG--------------------LLDPN
Tc_Snp           58 DFCTELTGIQQHQVDNGVPLQACLLLFSRWVAEKMSLYDMDFPNGESQ----------AT
Dm_Snp           59 AYCTELTGIQQKTVDSGMPLRTAIVMFNEWLRNEMRARNLTLPKMNKS---------NIL
Urchin_1         57 KFCSQLTNISQKMVDKADEFPTVLEKAERWMRQKG--------------------LGSKH
Urchin_2         56 DFCTELTGISQEQVENGVPLFICLNKFTSWLKKIESEKNLAYNRS-TD---------DTR
Urchin_3         55 EFCTRLTGITPDMVKGQPTFADTLKLFHVWMEKEG--------------------LVDPE
```

```
Ce_eri1         96 SRFAFVTDGPHDMWKFMQFQCLLSNIRMPHMFR-SFINIKKTFKEKFNGL--IKGNGKSG
Ce_M02B7.2      98 GNFAFVTCGDWDLKVALPSEAKFKNIEIPEYFN-QWINVKKAYAEHTN------HFAK-G
Hs_3hExo        97 K-YSLLTDGSWDMSKFLNIQCQLSRLKYPPFAK-KWINIRKSYGNFYK-----VPRSQTK
Hs_NP542394    111 KLCAFVTWSDWDLGVCLEYECKRKQLLKPVFLN-SWIDLRATYKLFYR------RKPK-G
Hs_Pint1        95 VKSIFVTCGDWDLKVMLPGQCQYLGLPVADYFK-QWINLKKAYSFAMG------CWPKNG
Tc_Snp         108 KTCAFATWSDWDLGTCLRKECIRKNIRIEKMYR-KWIDIRALFKRYIR------RPFI-G
Dm_Snp         110 GNCAFVTWTDWDFGICLAKECSRKGIRKPAYFN-QWIDVRAIYRSWYK------YRPC-N
Urchin_1        97 S-FAIATDCSLDMDLYLKLQCLVSEIPYPQYAK-EWVNISKVFANLYK-----TKR--LP
Urchin_2       106 KLCTFVTWSDWDLSVCLRYECQRKQLYKPSALN-HWIDARAVYRNFYQ------RKPK-G
Urchin_3        95 VKFIFVTCGDWDLKTMFPSQCAHFELPYHSYFR-RWLNIKKAFATVTS------HYPK-G

Ce_eri1        153 IENMLERLDLSFVGNKHSGLDDATNIAAIAIQMMKLK
Ce_M02B7.2     150 MTQLLAIYKLQHQGRLHSGIDDVANICEIVRCLGRDG
Hs_3hExo       150 LTIMLEKLGMDYDGRPHCGLDDSKNIARIAVRMLQDG
Hs_NP542394    163 LSGALQEVGIEFSGREHSGLDDSRNTALLAWKMIRDG
Hs_Pint1       148 LLDMNKGLSLQHIGRPHSGIDDCKNIANIMKTLAYRG
Tc_Snp         160 LAGALAELGLTFEGTEHCGLHDARNTARLVGKMVDKG
Dm_Snp         162 FTDALSHVGLAFEGKAHSGIDDAKNLGALMCKMVRDG
Urchin_1       148 LRAMLDSTGLAFIGQPHRGIDDARNIARVALQLIEDG
Urchin_2       158 LSGALQEVGIEFAGRQHSGLDDAKNTAILVWRMIQDG
Urchin_3       147 MMQMLERLNIPHVGRHHSGIDDCRNIANILQALALKR
```

Sid-1-related proteins

```
Tc_A             1 YLWHILSIAIFYSIPVVQLVITYQRVVNR---TGDQDMCYYNFLCANPAFGLSDFNHIFS
Tc_B             1 YLWQILNVGLFYIIPVIQLVVTLQSFLIQ---TGDFDLCYYNFRCANPLWIISDFNHVFS
Tc_C             1 YLYNVLTVALFYGLPVIQLVVTYQRALNE---TGQQDLCYYNFLCAHPLGVISDFNHVFS
Ce_sid1          1 LHGQMLQYPVAIILPVLMHTAIEFHKWTTSTMANRDEMCFHNHACARPLGELRAWNNIIT
Ce_tag130        1 YKIALAIIGIFYNITVLQLIISKAGSLRQ---SGDLDECTFNFQCARPLWYFVAFNNVVS
Ce_Y37H2C1       1 KYFNFLFFQLFGSILPALTTLFQNRLQKAN--KTNLDTCYLNYRCSLNVIGFNSFNSMTS
Am_sid1          1 YLYYLITVAIFYTLPAVQLLITYQHVLHV---TGNQDMCYYNFLCAHPFQALSDFNHVFS
Hs_sidT1         1 YFWNIITIAVFYALPVIQLVITYQTVVNV---TGNQDICYYNFLCAHPLGVLSAFNNILS
Hs_sidT2         1 YFWNIATIAVFYALPVVQLVITYQTVVNV---TGNQDICYYNFLCAHPLGNLSAFNNILS
Bm_3022          1 YFWGALTLAVVYALPVVQLLLTYQRMVFQ---TGDQDLCYYNFLCAHPLGTLSDFNHVFS
Bm_3026          1 YLWTVLTVSVFYTLPVLQLVVTYQRLLNQ---SGNQDLCYFNFFCAHPLMMLSDFNHVFS
Bm_2839          1 YLHTLYTVAVFYALPVLQFVAAFQVMLNI---SGSLDMCYYNFLCAHPAGGLSDFNHVFS

Tc_A            58 NVGYIIVGILFLGVVLHRQTKIPNSST---------GIPVHYGVYYAMGIALIIEGILSA
Tc_B            58 NIGYILMGIVFSINVFYRHFYSPPLTT---------GVPANYGVFYAMGAALIMEGVLSG
Tc_C            58 NSGYVLLGLLFLGITYRREITHKDLNFER-----QYGIPQHYGMFYAMGVALIMEGVLSG
Ce_sid1         61 NIGYTLYGAIFIVLSICRRGRHEYSHV--------FGTYECTLLDVTIGVFMVLQSIASA
Ce_tag130       58 NGGYVYFGTLIIVMNYCRERSFRRLFAVQPTLAERYGLPQHSGLMTAIGLAVIMEGISSA
Ce_Y37H2C1      59 ASSLAVLGIINLIIVFRKQIFRYEIPRFPT----SRGIQQRDAPKIVCLLGLVALGVYSA
Am_sid1         58 NIGYIMLGFLFIFLTSFREHNEFDKEKNK-----CYGIPQHYGLFYAMGTALIMEGILSG
Hs_sidT1        58 NLGHVLLGFLFLLIVLRRDILHRRALEAKDIFAVEYGIPKHFGLFYAMGIALMMEGVLSA
Hs_sidT2        58 NLGYILLGLLFLLIILQREINHNRALLRNDLCALECGIPKHFGLFYAMGTALMMEGLLSA
Bm_3022         58 NVGYVLLGAVFAGQVRFRQVKSRQRPE-------NLGIPQHYGLLYSMGLALSMEGLLSA
Bm_3026         58 NLGYVVLGALFLLQVWRRQRIMRNEPEEKK----QKGIPQHFGLLYAMGVALISEGFLSA
Bm_2839         58 NLGYLLLGALFMLQLQRRKRNRKRAPR-HE----EYGIPAHYGLLSSLGAAMMVVALLSA

Tc_A           109 CYHICPSQSNYQFDTSFMYVMAVLCMIKLYQNRHPDVNATAYATFTVLGMAIFLAMIGIL
Tc_B           109 CYHLCPNETNFQFDTSFMYVMIVLCLVKLYQNRHPDVTPTAYTTFSILGATILCGTIGIV
Tc_C           113 SYHVCPNTANFQFDSSFMYVMAVLCMVKLYQNRHPDINATAYATFGVLAVAILLGMIGIL
Ce_sid1        113 TYHICPSDVAFQFDTPCIQVICGLLMVRQWFVRHESPSP-AYTNILLVGVVSLN------
Ce_tag130      118 TYHVCPNNINYQFDTALMYVIGMLGKLKIWSLRHPDMVVSAYHAFGFLGVFLMAAIAGVY
Ce_Y37H2C1     115 ILNNCQQKSMLQFYDYTILWLQYSALLWIYSKRHG-MFVVDSSLRLIIKIVFSICSIGFT
Am_sid1        113 SYHVCPNRSNFQFDSSFMYIITVLCMIKIYQTRHPDINARASVTFAMLAFIIFINLMGVL
```

```
Hs_sidT1     118 CYHVCPNYSNFRFDTSFMYMIAGLCMLKLYQTRHPDINASAYSAYASFAVVIMVTVLGVV
Hs_sidT2     118 CYHVCPNYTNFQFDTSFMYMIAGLCMLKLYQKRHPDINASAYSAYACLAIVIFFSVLGVV
Bm_3022      111 CYHLCPNKMNFQFDSSFMYVIAVLVTLKLYQNRHSDIIPSAHSTFMILAVIMTIGLFGIL
Bm_3026      114 AYHVCPNSMNFQFDTSFMYVTSALCMVKIYQSRHPDINARAHATFGVLALIIFIGLVGVL
Bm_2839      113 SYHVCPNSLNFQFDTAFMYVLAVLCMVKIYQSRHPDINARAHATFGVLAVFIALVVWGVL

Tc_A         169 NG-SLTVWIVFVVI-YSLLCAYISFKIYFISFVFDGFKQLKQSLKSSNKV------EAIA
Tc_B         169 FK-APPVFIVFVTIAYLVLLIYASLNIYHFGTARNFLR--RCCLRNSEVP------RPIQ
Tc_C         173 EG--NLYFWIVFTIIYLLSCFYLSIQIYYMGCWKLDAGLAMRVWRICVYEFWSGPLNVIK
Ce_sid1      166 ------FLISAFSKTSYVRFIIAVIHVIVVGSICLAKERSLGSEK---------------
Ce_tag130    178 VH--NMIFWALFSIIYIASMLLVSLEFYFKGIWTLNLRELRNSIRLSWVSS--RHLSCVV
Ce_Y37H2C1   174 GYFAYKYFYERPSGLQSHQWICTPLSSQQK-VLEDDKNVKRRPLN---------------
Am_sid1      173 NG--SIYFWILFTITHLLTCLFMTIQIYYMGRWKFRA-LLTRVLQVILYS--SGIRYLFR
Hs_sidT1     178 FGKNDVWFWVIFSAIHVLASLALSTQIYYMGRFKIDLGIFRRAAMVFYTD---CIQQCSR
Hs_sidT2     178 FGKGNTAFWIVFSIIHIIATLLLSTQLYYMGRWKLDSGIFRRILHVLYTD---CIRQCSG
Bm_3022      171 HP--SAGFAASFTLLHLGACLVLTLKIYYAGRFKMDRRVLLRAYAHVAARG----WRSLL
Bm_3026      174 NA--NVYFWVAFTALHLLTCFFITFQIYYLGRFKLDMGWVRAASRGAALR----------
Bm_2839      173 GG--GPLFWSVFTVLHVFTFLLLSLRIYYVGQFRLEKSSLAVAARGLRAR----------

Tc_A         221 PIRKSRFALLVIANIINYAMLITGLCLYN-TGVTDFGTFLLGLLMGNSVLYAVFYTGMKL
Tc_B         220 SPNTHRWWLLLLAITVNILLYGLGLILFYHTKTIDFATFILQILAGNAFLYTVVYTCMKI
Tc_C         231 PIHKARMCLLIIANLCNWGMAFWG---VYKHQ-KDFALFLLAIFMGNTLLYFSFYIVMKI
Ce_sid1      205 --LKTRFFIMAFSMGNFAAIVMY-----LTLSAFHLNQIATYCFIINCIMYLMYYGCMKV
Ce_tag130    234 PAYKARFFVILLLNIANTAVVVYG----LEAHPKDFLSFLLIPFIGNLFIYIIYYILMKM
Ce_Y37H2C1   218 --SKMSYVFISIGYAIICSIVTFL------HSGISVHSAIYFSAKGQLVIYLFFYIIQKI
Am_sid1      228 PLYIGRFFMLVIANLWNIALAVIG---NIYQE-KNFATFLLAILMSNLILYTTFYIIMKI
Hs_sidT1     235 PLYMDRMVLLVVGNLVNWSFALFG---LIYRP-RDFASYMLGIFICNLLLYLAFYIIMKL
Hs_sidT2     235 PLYVDRMVLLVMGNVINWSLAAYG---LIMRP-NDFASYLLAIGICNLLLYFAFYIIMKL
Bm_3022      225 PAHPYRAGLLGLANLANWSLAGYS---VYSHHNTDLARQLLAILMGNAILYTMFYMVMKL
Bm_3026      222 ---PSRGLMLLLANLINWGLAGYG---VAQHS-RDFASHLLLVLMTNLFLYTLFYIVMKL
Bm_2839      221 PLYTPRLVMLLIANAANWGFAIYG---LLTHA-GDIATHLLNVLLCNTLLYIVFYVLMKL

Tc_A         280 VN--------GERICFEAIIYGLLAIAAWATAAVYFLDNAT--LWTVTPAESRQWNQECI
Tc_B         280 KCTSVRECTCSEKICAQAIIYGFLALVTWVLAGVFFFTEAS--KWTESPAQSRQLNKQCI
Tc_C         287 IN--------KERVNKLSLFFLSLSVLCAISAMYFFLNKSI--SWSRTPAQSRQFNQECK
Ce_sid1      258 LH--------SERITSKAKLCGALSLLAWAVAGFFFFQDDT--DWTRSAAASRALNKPCL
Ce_tag130    290 IY--------REKIPKRAIALLFAAVISWTCAGILFNQRVS--DWSKMPAISRELNKPCI
Ce_Y37H2C1   270 RFEWSS----FTFSYTIRCIFLIVFLVLFQTLNRYFANFASPFNFYESPALSRELNSECI
Am_sid1      284 CH--------KERILLQPCIYIVLSIVFWAAALYFFINKTI--SWELTSAQSRHYNKPCE
Hs_sidT1     291 RS--------SEKVLPVPLFCIVATAVMWAAALYFFFQNLS--SWEGTPAESREKNRECI
Hs_sidT2     291 RS--------GERIKLIPLLCIVCTSVVWGFALFFFFQGLS--TWQKTPAESREHNRDCI
Bm_3022      282 VN--------RERILARTWMYCILAHVAWFLALRLFLDSKT--KWSETPAQSRQHNAPCS
Bm_3026      275 LH--------RETITCYTWVFIVLTYSSWAGSSYFYLDQNT--NWALSPAQSRERNAACS
Bm_2839      277 LH--------GERIRWYSWCFLAAAAACWVPALYFFTSGST--DWSATPARSRHRNHECR

Tc_A         330 VMSFYDKHDVWHLLSAPALYLTFMFLLSLDDDL
Tc_B         338 FADFYDSRDLWHFFSSLALYFTFMYLLCIDDNL
Tc_C         337 LLRFYDFHDIWHFLSAIGMFFTFMVLLTLDDDL
Ce_sid1      308 LLGFFGSHDLWHIFGALAGLFTFIFVSFVDDDL
Ce_tag130    340 FLNFYDNHDLWHLSSAFAIFFSFTAINVIDDDL
Ce_Y37H2C1   326 IPG-IDWNNVRHYNAVITCSFFILLLDYIDINI
Am_sid1      334 LLHFFDSHDIWHFLSALAMFFSFMVLLTLDDDL
Hs_sidT1     341 LLDFFDDHDIWHFLSATALFFSFLVLLTLDDDL
Hs_sidT2     341 LLDFFDDHDIWHFLSSIAMFGSFLVLLTLDDDL
Bm_3022      332 SLSFYDTHDLWHGVSAAALFLSFNMLLTMDDAL
Bm_3026      325 VLRLFDAHDAWHAMSAVAMFLSFNMYLTLDDGL
Bm_2839      327 VLQFYDSHDLWHMLSAAALYFTFNVMLTWDDGL
```
