# Supplementary figures and images for: Exploring systemic RNA interference in insects: a genome-wide survey for RNAi genes in Tribolium
Source: Genome Biol. 2008 Jan 17;9(1):R10. doi: 10.1186/gb-2008-9-1-r10 (PMC2395250; doi:10.1186/gb-2008-9-1-r10)

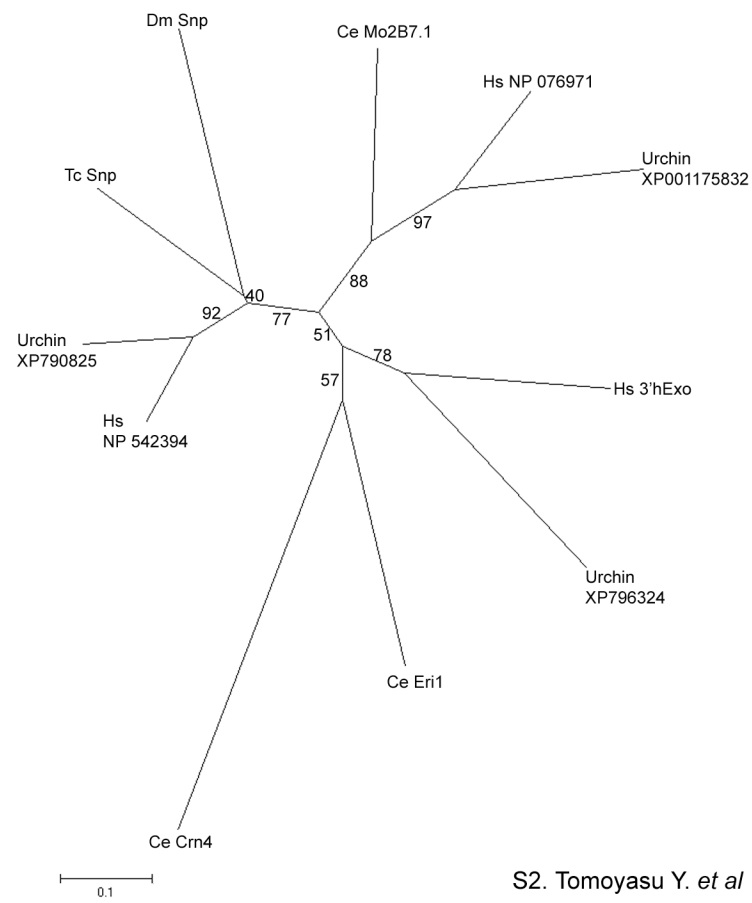

S2. Tomoyasu Y. *et al*

Supplement: Additional data file 2 — Phylogenetic tree for Eri-1-like nucleases including C. elegans Crn-4. [file gb-2008-9-1-r10-S2.pdf]

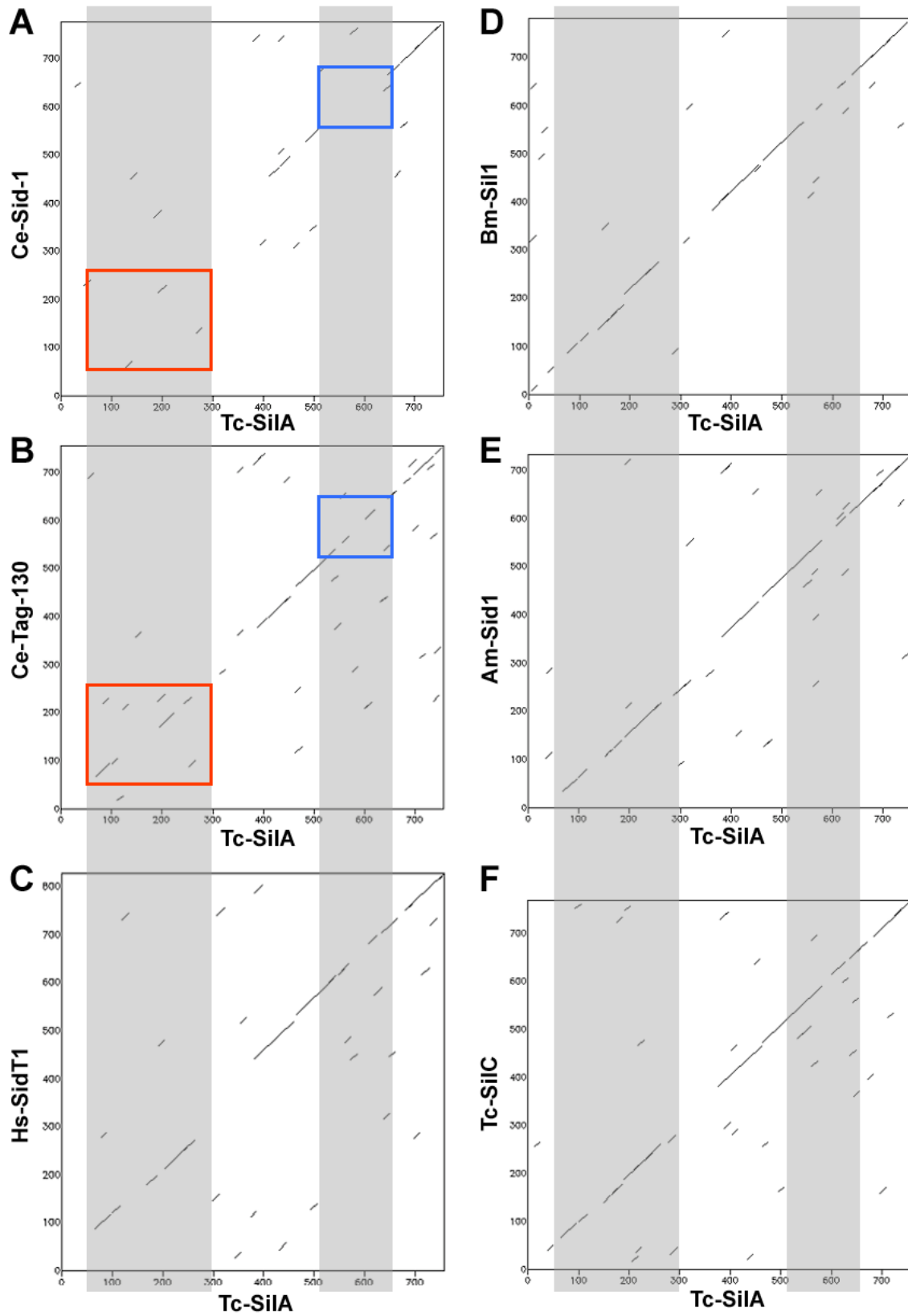

Supplement: Additional data file 5 — Dot-matcher alignments of Tc-SilA protein with Sid-1-like proteins from various organisms. Conservation between two proteins is visualized as a diagonal line. Tc-SilA does not show high conservation with Ce-Sid-1 in the amino-terminal extracellular region (A, red box), but shows conservation with Ce-Tag130 (B, red box). Additional conservation is seen in the carboxy-terminal transmembrane domains (B, blue boxes), which is lacking in Ce-Sid-1(A, blue box). These conserved domains are seen in all Sid-1-like proteins examined (B-F), except Ce-Sid-1 (A). [file gb-2008-9-1-r10-S5.pdf]

**A**

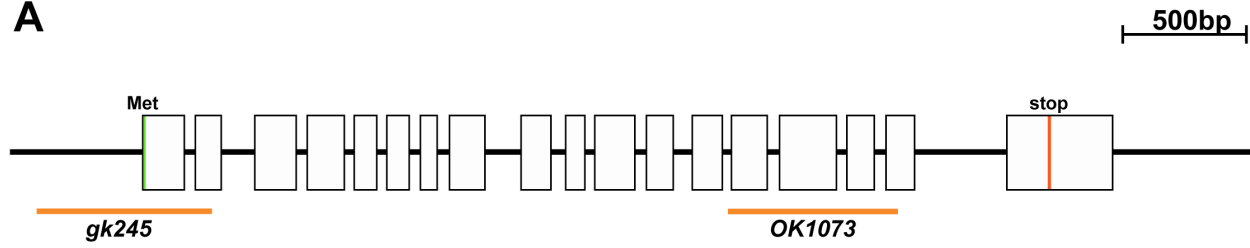

**B**

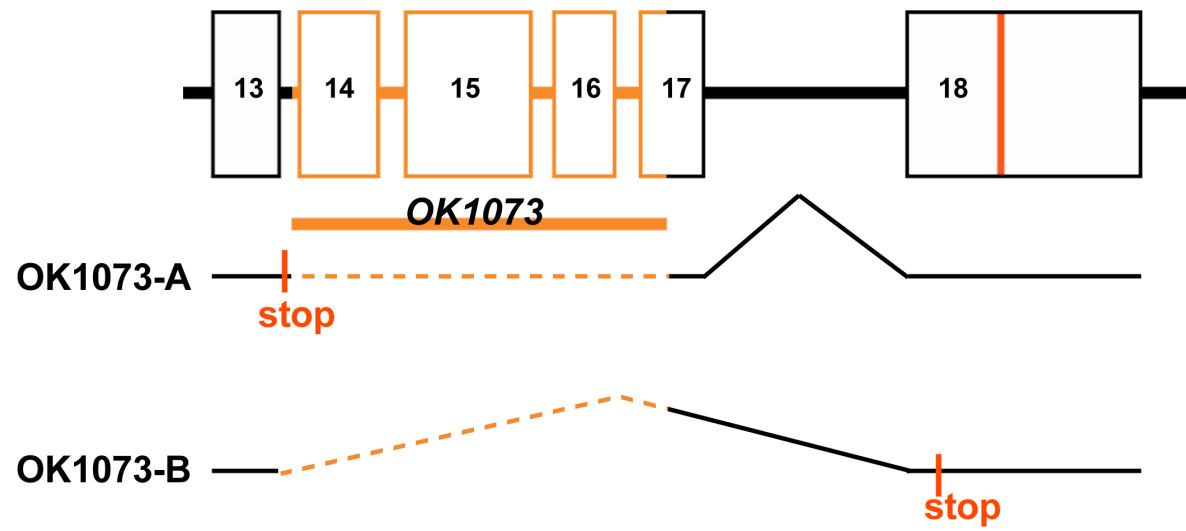

Supplement: Additional data file 6 — (A) tag-130 gene exon/intron structure. The regions deleted in tag-130gk245 and tag-130OK1073 are indicated with orange bars. (B) An enlargement of the OK1073 deleted region and schematic diagrams of two mRNA forms detected in tag-130OK1073 mutants. The deleted region is indicated in orange. In isoform OK1073-A, which is the more abundant of the two forms, the remaining portion of intron 13 is not spliced out and is juxtaposed with the remaining portion of exon 17. Intron 13 contains a stop codon in this reading frame, which should cause truncation of the protein. In the other isoform (OK1073-B), the remaining portion of intron 13 is spliced out along with the remaining portion of exon 17 (and intron 17), juxtaposing exon 13 with exon 18. This changes the reading frame in exon 18, and should also result in premature truncation of the protein. [file gb-2008-9-1-r10-S6.pdf]
