# Supplementary material for: Exploring systemic RNA interference in insects: a genome-wide survey for RNAi genes in Tribolium
Source: Genome Biol. 2008 Jan 17;9(1):R10. doi: 10.1186/gb-2008-9-1-r10 (PMC2395250; doi:10.1186/gb-2008-9-1-r10)
Supplement: Additional data file 3 — sil gene expression profile in Tribolium. [file gb-2008-9-1-r10-S3.pdf]

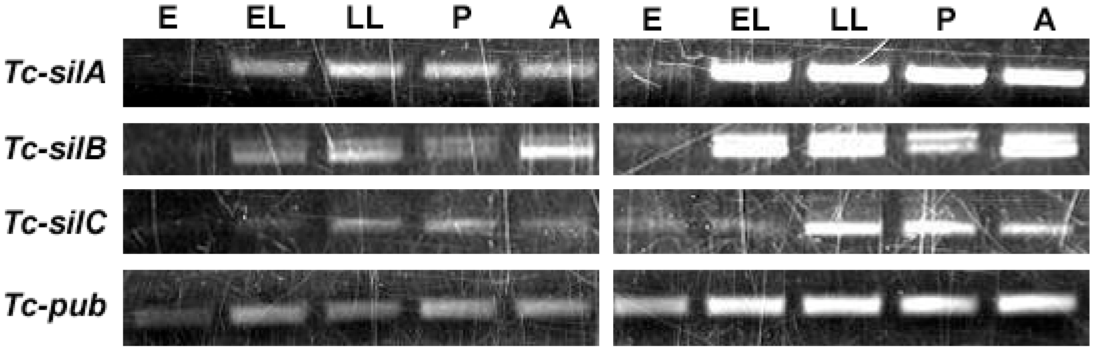

E:embryonic stage, EL:early larval stage, LL:late larval stage
P:pupal stage, A:adult stage

silA and silB: 24 cycles (left) and 26 cycles (right)
silC and polyUbiquitin: 26 cycles (left) and 28 cycles (right)

S3 Tomoyasu Y. *et al.*
