# Supplementary material for: Exploring systemic RNA interference in insects: a genome-wide survey for RNAi genes in Tribolium
Source: Genome Biol. 2008 Jan 17;9(1):R10. doi: 10.1186/gb-2008-9-1-r10 (PMC2395250; doi:10.1186/gb-2008-9-1-r10)
Supplement: Additional data file 4 — Blue boxes indicate conserved portions of the amino-terminal extracellular domain shown in Figure 6. Amino acids making up predicted transmembrane regions for each protein are shown in orange, while the 11 predicted transmembrane domains of Tc-SilA protein are denoted with orange bars. The region corresponding to TM2 to TM11 (delimited by green arrows) was used for phylogenetic analysis. [file gb-2008-9-1-r10-S4.pdf]

```
Ce_tag130    1 ...................MRTSQAIFILIFLDSVRNQSPQVIPAKWDVVYEKETGHNMS.
Tc_silA      1 ...........VQFEPRAMIAAAGLLLLVPLADCAHIASLNIEQHQGNYSQVMPFLFNQTT
Hs_sidT1     1 MRGCLRLALLCALPWLLLAASPGHPAKSPRQPPAPRRDPFDAARGADFDHVYSGVVNLST
Ce_sid1      1 ...MIRVYLIILMHLVIGLTQNNSTTPSPIITSSNSSVLVFEISSKMKMIEKKLEANTVH

Ce_tag130   42 LTVFRF...QVKEQYSVARIIMSCNESTEHNPLLAVFREKLAILSLQVPLIVDN......
Tc_silA     51 EHVLVF...PTSDSIYPYRVKAWSSGAKLASPVLVVVRQEREVISWQVPFVVDTTMKDGV
Hs_sidT1    61 ENIYSFNYTSQPDQVTAVRVYVNSSSENLNYPVLVVVRQQKEVLSWQVPLLFQG.LYQRS
Ce_sid1     58 VLRLEL...DQSFILDLTKVAAEIVDSSKYSKEDGVILE.VTVSNGRDSFLLKLPTVYPN

Ce_tag130   93 YEYSQVARTLCP................FTEYKEGEAFTVEVTSSRPVHYNFRAE....L
Tc_silA    108 VHFHNTSRTLCHNDMPRIAKAKATSRILPIQLSQNFIIALSTSSLANVDISVMVE....E
Hs_sidT1   120 YNYQEVSRTLCPS........EATN..ETGPLQQLIFVDVASMAPLGAQYKLLVT....K
Ce_sid1    114 LKLYTDGKLLNPLVEQDFGAHRKRHRIGDPHFHQNLIVTVQSRLNADIDYRLHVTHLDRA

Ce_tag130  133 VQNFYLYNNSQRLVTASASE.....PVYLRYDIPGDVDS.VAVHLDSNSTICMTVSVQKI
Tc_silA    164 ERDFYLQEGRPYEVSVSPSE.....SKYYYYKFHDKKNTSAMIEINSDDDVCLTVSIQDS
Hs_sidT1   166 LKHFQLRTNVAFHFTASPSQ.....PQYFLYKFPKDVDSVIIKVVSEMAYPCSVVSVQNI
Ce_sid1    174 QYDFLKFKTGQTTKTLSNQKLTFVKPIGFFLNCSEQNISQFHVTLYSEDDICANLITVPA

Ce_tag130  187 GCPVFDLPDNVNSMGLHQTMTTSATIPVE.....KSRMSSFYVVFVVNTNDDLCSEILSI
Tc_silA    219 FCPVFDLDKDITYEGKYQTINRKGGMTIRQ....REFPDGFFLVFVAKADNYQCSQKHSV
Hs_sidT1   221 MCPVYDLDHNVEFNGVYQSMTKKAAITLQK...KDFPGEQFFVVFVIKPEDYACGGSFFI
Ce_sid1    234 NESIYDRSVISDKTHNRRVLSFTKRADIFFTETEISMFKSFRIFVFIAPDDSGCSTNTSR

                                                   TM1
Ce_tag130  242 KPNKP....TKFPLRMKSFNVTIESSMKIFDYTIPIVFWACILLLVTIVVFVYHYFDGIW
Tc_silA    275 LLVEHRKQHLILANRTSTITFTINKGINGKEYEIASLATLGALLSFCIVSTIMIFAFTRW
Hs_sidT1   278 QEKENQ...TWNLQRKKNLEVTIVPSIKESVYVKSSLFSVFIFLSFYLGCLLVGFVHYLR
Ce_sid1    294 KSFNEKKKISFEFKKLENQSYAVPTALMMIFLTTPCLLFLPIVINIIKNSRKLAPSQSNL

Ce_tag130  298 ERRFVSRAYTHLEDNAQEQRIRDFY.................................DF
Tc_silA    335 GTISKFRPSGDELDADWEE.........................................
Hs_sidT1   335 FQRKSIDGSFGSNDGSGNMVASHPIAASTPEGSNYGTIDESSSSPGRQMSSSDGGPPGQS
Ce_sid1    354 ISFSPVPSEQRDMDLSHDEQQNTSS.................................EL

                                                               TM2
Ce_tag130  325 QRMSEDDDLKDYDLLTDCQDMMVVRAKASLTVADLSMTPYEERELKYDVYKIALAIIGIF
Tc_silA    354 ..........PPEPPITRELKHELLSRQALTVNLLARAPEKDK.RRSYNYLWHILSIAIF
Hs_sidT1   395 DTDSSVEESDFDTMPDIESDKNIIRTKMFLYLSDLSRKDRRIVSKKYKIYFWNIITIAVF
Ce_sid1    381 ENNGEIP.AAENQIVEEITAENQETSVEEGNREIQVKIPLKQDSLSLHGQMLQYPVAIIL

                                                    TM3
Ce_tag130  385 YNITVLQLIISKAGSLRQSGDLDECTFNFQCARPLWYFVAFNNVVSNGGYVYFGTLIIVM
Tc_silA    403 YSIPVVQLVITYQRVVNRTGDQDMCYYNFLCANPAFGLSDFNHIFSNVGYIIVGILFLGV
Hs_sidT1   455 YALPVIQLVITYQTVVNVTGNQDICYYNFLCAHPLGVLSAFNNILSNLGHVLLGFLFLLI
Ce_sid1    440 PVLMHTAIEFHKWTTSTMANRDEMCFHNHACARPLGELRAWNNIITNIGYTLYGAIFIVL

                                       TM4
Ce_tag130  445 NYCRERSFRRLFAVQPTLAERYGLPQHSGLMTAIGLAVIMEGISSATYHVCPNNINYQFD
Tc_silA    463 VLHRQTKIP.........NSSTGIPVHYGVYYAMGIALIIEGILSACYHICPSQSNYQFD
Hs_sidT1   515 VLRRDILHRRALEAKDIFAVEYGIPKHFGLFYAMGIALMMEGVLSACYHVCPNYSNFRFD
Ce_sid1    500 SICRRGRHE........YSHVFGTYECTLLDVTIGVFMVLQSIASATYHICPSDVAFQFD

                TM5                      TM6                        TM7
Ce_tag130  505 TALMYVIGMLGKLKIWSLRHPDMVVSAYHAFGFLGVFLMAAIAGVY..VHNMIFWALFSI
Tc_silA    514 TSFMYVMAVLCMIKLYQNRHPDVNATAYATFTVLGMAIFLAMIGIL..NGSLTVWIVFVV
Hs_sidT1   575 TSFMYMIAGLCMLKLYQTRHPDINASAYSAYASFAVVIMVTVLGVVFGKNDVWFWVIFSA
Ce_sid1    552 TPCIQVICGLLMVRQWFVRH.ESPSPAYTNILLVGVVSLNFLISAF..SKTSYVRFIIAV
```

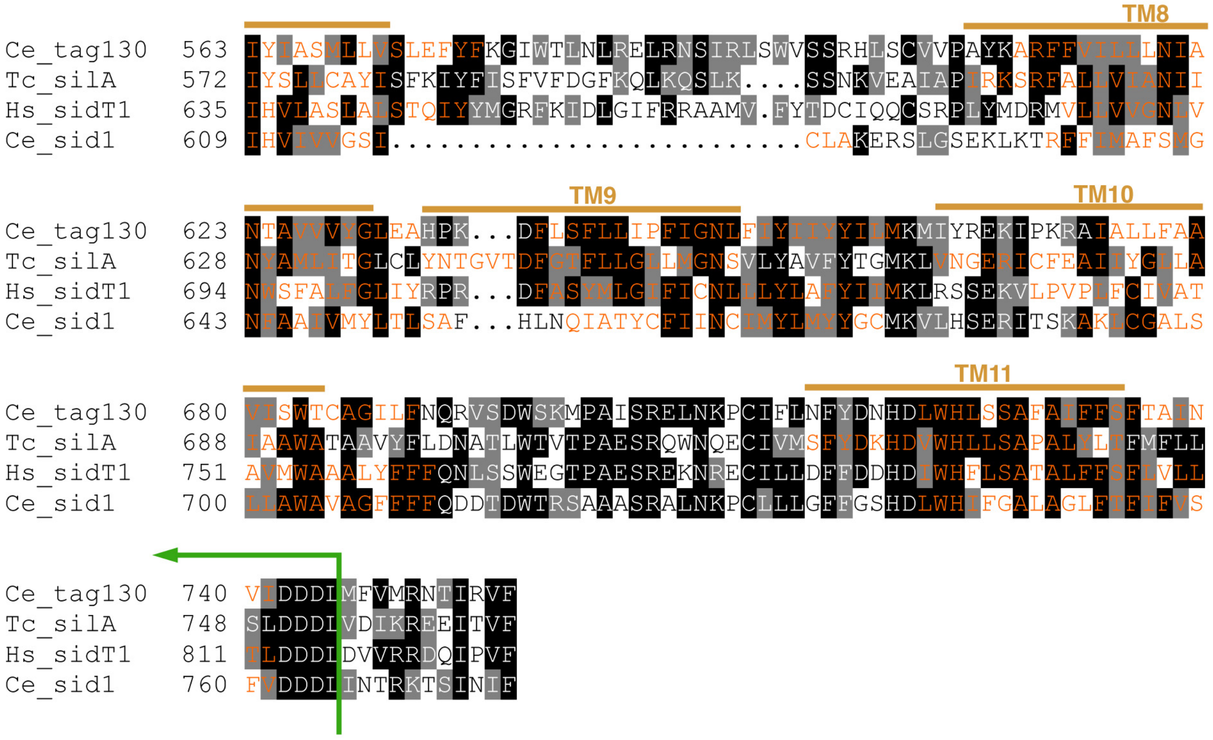

S4. Tomoyasu Y. ***et al.***
