# Supplementary material for: Exploring systemic RNA interference in insects: a genome-wide survey for RNAi genes in Tribolium
Source: Genome Biol. 2008 Jan 17;9(1):R10. doi: 10.1186/gb-2008-9-1-r10 (PMC2395250; doi:10.1186/gb-2008-9-1-r10)
Supplement: Additional data file 7 — GLEAN gene number and corresponding gene names. [file gb-2008-9-1-r10-S7.pdf]

Table S7: *Tribolium* gene names and gene ID

Tc Gene Name	Tc Gene Number	Linkage Group
Tc-Dicer-1	01750	unknown
Tc-Dicer-2	01108	2
Tc-Drosha	16208	unknown
Tc-Pasha	15332	6
Tc-Loquacious	11666	9
Tc-R2D2	08716	7
Tc-C3PO	07013	4
Tc-Argonaute-1	05857	8
Tc-Argonaute-2a	11525	unknown
Tc-Argonaute-2b	13762	5
Tc-Argonaute-3	08511	4
Tc-PIWI	08711	7
Tc-snipper	01174	2
Tc-SilA	11760	9
Tc-SilB	06161	8
Tc-SilC	15033	6
Tc-Rsd3	12168	9
