# Supplementary material for: Exploring systemic RNA interference in insects: a genome-wide survey for RNAi genes in Tribolium
Source: Genome Biol. 2008 Jan 17;9(1):R10. doi: 10.1186/gb-2008-9-1-r10 (PMC2395250; doi:10.1186/gb-2008-9-1-r10)
Supplement: Additional data file 8 — RNAi and miRNA components in Tribolium, Drosophila and C. elegans. [file gb-2008-9-1-r10-S8.pdf]

Table S8: RNAi and miRNA components in *Tribolium, Drosophila* and *C.elegans*

	Tribolium	***Drosophila***	***C. elegans****	**Remarks**
**dsRBM proteins**	R2D2<br>C3PO	R2D2	Rde-4	
	Loquacious	Loquacious		
	Pasha	Pasha		
**Dicer**	Dicer-1<br>Dicer-2	Dicer-1<br>Dicer-2	Dicer-1	While Tc-Dcr-1 apears orthologous to Dm-Dcr-1, the domain architecture of Tc-Dcr-1 is more similar to that of CeDcr1
	Drosha	Drosha		
**1ºArgonaute**	Argonaute-1	Argonaute-1	Alg-1<br>Alg-2	
	Argonaute-2a<br>Argonaute-2b	Argonaute-2	Rde-1<br>Ergo	
	Argonaute-3	Argonaute 3	Prg-1<br>Prg-2	
	PIWI	PIWI<br>Aubergine		
**2ºArgonaute**			SAGO-1<br>SAGO-2<br>PPW-1<br>PPW2	
**RdRP**			Ego-1<br>RRF-1<br>RRF-3	
**Eri-1 like nuclease**			M02B7.2	
			Eri-1	
	Snipper	Snipper		
**Sid-1-like**	SilA<br>SilB<br>SilC		Y37H2C1<br>Sid-1<br>Tag-130	The orthology between the Tc Sil proteins and the Ce Sid like proteins is not resolved

* *C.elegans* column does not show the whole inventory. Only some representatives are shown.
