# Supplementary material for: Exploring systemic RNA interference in insects: a genome-wide survey for RNAi genes in Tribolium
Source: Genome Biol. 2008 Jan 17;9(1):R10. doi: 10.1186/gb-2008-9-1-r10 (PMC2395250; doi:10.1186/gb-2008-9-1-r10)
Supplement: Additional data file 9 — Primers used for dsRNA synthesis. [file gb-2008-9-1-r10-S9.pdf]

Table S9

Candidate Gene	Primers	Sequence	Product Size
	Dicer1 F2	AGGCTCTAATCGGGGCGTATCTGA	
Dicer-1	Dicer1 R2	GTTGCGGAAGTAGCGGTGGAAGC	473bp
	Dicer2 F1	TGCGCCATCCACCCGTTTTCTGC	
Dicer-2	Dicer2 R1	TTATCCCGCTTGGCTTACCTTCTA	413bp
	Ago1 F1	CAGAGTTTGGGCCATCGCTTGTTT	
Argonaute-1	Ago1 R1	TATTGTCGCCGGCTGGAGGATGA	476bp
	Ago2a F1	GCCGCCGACTCTGCTGTATGC	
Argonaute-2a	Ago2a R1	TGGCGATTATTTGCGGGTTGAGTT	425bp
	Ago2b F2	TGGAGGGCGGACAGGAATAGGTTT	
Argonaute-2b	Ago2b R2	GCAGCCGCCAGCAGATGTTGT	580bp
sid like A*	sidA RCE R2	GTTGGGGATTTTGGTCTGCCGATGTAGTA	854bp
	sid1B F1	ACCGGAGATTTCGACCTGTGC	
sid like B	sid1B R1	CTGTCGTAAAAGTCCGCAAAAATG	944bp
	sid1C F1	GCACATCCTTTGGGGGTTATCAG	
sid like C	sid1C R1	GCGCTCAAAAAGTGCCAAATGT	929bp

* there is no forward primer for this clone since this is a 5'RACE product.
5' side of this clone begins with ACTACAAGTTCCATGATAAG
